# Revision of the Palaearctic species of the genus *Plateumaris* C. G. Thomson, 1859 (Coleoptera, Chrysomelidae, Donaciinae)

**DOI:** 10.3897/zookeys.1177.103214

**Published:** 2023-08-30

**Authors:** Elisabeth Geiser

**Affiliations:** 1 Natural History Museum, Burgring 7, 1010 Vienna, Austria Natural History Museum Vienna Austria

**Keywords:** Identification key, leaf beetles, new country records, new synonymies, reed beetles, revision, Systematics, taxonomy

## Abstract

Ten of the 27 species of *Plateumaris* Thomson (Chrysomelidae: Donaciinae) occur in the Palaearctic. Due to the intraspecific variation and the large distributions of some species, descriptions exist for at least 80 taxa plus five nomina nuda. The status of each valid species is clarified and the remaining 70 names are allocated as synonyms. New synonymies are *P.tenuicornis* Balthasar, considered a synonym of *P.consimilis* (Schrank), *P.sulcifrons* Weise as a synonym of *P.rustica* (Kunze), and *P.caucasica* Zaitzev as a synonym of *P.sericea* (Linnaeus). Two controversial synonyms are confirmed: *P.discolor* (Panzer) and *P.sericeasibirica* (Solsky) are both synonyms of *P.sericea*. Finally, *P.obsoleta* Jacobson is a synonym but at present it is not possible to decide whether it belongs to *P.shirahatai* Kimoto or to *P.sericea*. Forty-one new country records are added, compared with the Catalogue of Palaearctic Coleoptera published in 2010; 28 records are based on recently published records and 13 are first records for a specific country.

## Introduction

The genus *Plateumaris* Thomson, 1859 belongs to the subfamily Donaciinae (reed beetles) which is part of the beetle family Chrysomelidae (leaf beetles). This family includes more than 37,000 (probably at least 50,000) species in more than 2,500 genera, making up one of the largest beetle families (Jolivet et al. 1988). The Donaciinae comprise approximately 180 species belonging to six genera (Geiser 2015). Seventeen species of *Plateumaris* occur in the Nearctic region (Askevold 1991) and ten species in the Palaearctic region. Their distribution area extends from 30° north latitude to the Arctic Circle (pers. obs.).

Most life stages in Chrysomelidae (larvae and imagines) are terrestrial; however, the larvae of the Donaciinae develop submerged on roots of aquatic plants. These larvae breathe by tapping the aerenchyma of the plant with two hollow abdominal hooks which are connected to their tracheal system. Therefore, they can stay permanently under water. Monophyly of Donaciinae is supported by these special morphological and physiological adaptions as well as by molecular data (Kölsch and Pedersen 2008; Sota et al. 2008). These molecular analyses also revealed that the genus *Plateumaris* is a monophylum within the Donaciinae.

This number of ten species in the Palaearctic is not without controversy; in fact, different authors recognise nine to 19 species in the region (Table 1). This problem became virulent when I was editing the Donaciinae for the update of the catalogue of Palaearctic Chrysomelidae (Löbl and Smetana 2010; Silfverberg 2010), where the *Plateumaris* species are listed. To decide how many species occur in the Palaearctic region and what are their valid names, it became clear that a major revision was necessary. Simultaneously, several comprehensive works were published on Palaearctic Donaciinae, e.g., the ‘Identification Key on Palaearctic Chrysomelidae’ by Warchałowski (2010) and the book by Bieńkowski (2014) on the Russian Donaciinae, where almost all the Palaearctic *Plateumaris* species were treated. Hayashi (2020) dealt only with the Japanese Donaciinae that include half the Palaearctic *Plateumaris* species; his text complements Bieńkowski’s book. Finally, the molecular analyses of Hayashi and Sota (2014) are an invaluable help with systematic revisions.

A revision of the Palaearctic species of *Plateumaris* was needed, not only for the Palaearctic catalogue (Bezděk and Sekerka in press; Geiser in press). Colleagues working in physiology or ecology, especially in applied limnology, or those who are trying to reconstruct the dynamics of postglacial resettlement and similar topics which are important to understand climate changes and threat to biodiversity, all rely on solid species delimitations.

Knowledge of species distributions is necessary for systematic revisions. Therefore, I evaluated records from museum specimens and the literature. For the catalogue (Geiser in press) I provided this information only in a very concise manner. A revision is a good place to publish the detailed data and also provides an opportunity to explain the significance of a new record. For systematic revisions it is mandatory to study the first description of the taxa concerned. Many of these descriptions are in Latin or at least begin with Latin text. To make my arguments more understandable a companion article in this issue (Geiser and Geiser 2023) is published, where translations are provided of many original descriptions into English.

## Materials and methods

For this revision, approximately 1500 adult specimens including six type specimens from 16 museum collections were examined. These museums are indicated below, together with other museums which are cited as depositories.

### Museum acronyms

**BMNH** The Natural History Museum [formerly British Museum (Natural History)], London, UK (Michael Geiser, Maxwell V.L. Barclay, Keita Matsumoto, Dmitry Telnov)

**HNHM**Hungarian Natural History Museum, Budapest, Hungary (Ottó Merkl †, Tamás Németh)

**HNSA**Haus der Natur, Salzburg, Austria (Patrick Gros)

**KUEC** Entomological Laboratory, Faculty of Agriculture, Kyushu University, Fukuoka, Japan

**LUOMUS**Finnish Museum of Natural History, Helsinki, Finland (Jaakko Mattila)

**MNHN** Muséum national d’Histoire naturelle, Paris, France

**MSNV**Museo Civico di Storia Naturale, Verona, Italy (Mauro Daccordi, Roberta Salmaso, Leonardo Latella)

**NHMB**Natural History Museum, Basel, Switzerland (Matthias Borer, Christoph Germann, Eva Sprecher, Isabelle Zuercher)

**NHMW**Natural History Museum, Vienna, Austria (Manfred A. Jäch, Helena Shaverdo, Michaela Brojer, Harald Schillhammer, Matthias Seidel, Wolfgang Schönleithner †, Wolfgang Brunnbauer)

**NHRS**Swedish Museum of Natural History, Stockholm (Johannes Bergsten)

**NMEG**Natural History Museum, Erfurt, Germany (Matthias Hartmann)

**NMPC**National Museum (Natural History), Prague, Czech Republic (Lukáš Sekerka, Jiří Hájek)

**OMNH** Osaka Museum of Natural History, Japan

**RBINS** Royal Belgian Institute of Natural Sciences, Brussels, Belgium

**SDEI** Senckenberg German Entomological Institute, Müncheberg, Germany (Thomas Schmitt, Stephan Blank, Mandy Schröter)

**SMF**Senckenberg Museum Frankfurt, Germany (Andrea Hastenpflug-Vesmanis, Damir Kovac)

**SNMC**Slovak National Museum, Bratislava, Slovakia (Vladimir Janský, Martin Sečanský)

**ZFMK**Zoological Research Museum Alexander Koenig, Bonn, Germany (Dirk Ahrens, Karin Ulmen)

**ZIN**Zoological Institute of the Russian Academy of Sciences, St. Petersburg, Russia (Alexey Moseyko)

**ZMHB** Natural History Museum, Berlin, Germany (Johannes Frisch, Bernd Jaeger)

**ZSM**Bavarian State Collection of Zoology, Munich, Germany (Michael and Ditta Balke, Katja Neven, Martin Baehr †)

### Other abbreviations

**A1, A2**, … Number of an antennomere.

**ab.** aberratio.

**coll.** collection: the location where the specimen is stored.

**det.** determinavit: name of the person who identified this specimen.

**ex.** specimen(s).

**ex coll.** previous collection where the specimen was stored.

**f.** forma.

**ICZN** International Code of Zoological Nomenclature.

**leg.** legit: name of the person who collected this specimen.

**T1, T2**, … number of a tarsomere.

**var.** variatio.

**vid.** vidit: name of the person who confirmed the identification.

[] Text in square brackets [] are additions or comments by the author; in records data they do not form part of the label text tagged to the specimen.

[**leg.**] name of the person who probably collected this specimen.

[**det.**] name of the person who probably identified this specimen.

[**new in PalCat**] This indicates that the country mentioned was not listed in Silfverberg (2010) but is now listed in the update of the Palaearctic Catalogue (Geiser in press). This refers to already published records, and the data source is listed below.

[**first record**] If a country record was not published in detail until now, it is indicated as “first country record”. The same applies for a part of a country if it is treated as separate unit within a country, according to Bezděk and Sekerka (in press). These records were published in Geiser (in press) but only with the country or province abbreviation in most cases so the record details are given here.

// If original text is cited from different labels tagged to the pin of one specimen, the double slash indicates the text separation between these labels.

### Data sources

#### Museum specimens

Except for *Plateumarissericea*, it is very difficult to deliberately catch *Plateumaris* specimens in their habitat. Many voucher specimens were gained as by-catch in field studies motivated by other goals. The study of the variation of characters from many different sites of the whole distribution area is important for systematic revisions. Therefore, museum collections that have existed for a long time and where specimens from many different locations are stored are essential for such studies, but even smaller collections could provide a pleasant surprise to find some voucher specimens of a rare species from an interesting site.

The specimens in the museum collections were sorted in boxes with a species label. The compilation was usually done by non-specialists, who sorted the various collections donated to the museum into the overall collection, regardless of whether they were correctly identified or not. The vouchers of *P.bracata*, *P.consimilis*, *P.rustica*, and *P.sericea* were usually correctly identified. Specimens of other *Plateumaris* species are rare, and these were all examined in detail. Some of them already had identifier labels. If not, I tagged my label on the needle with the information “genus species Author det. E. Geiser Year”. I also did that when a specimen was misidentified, leaving the original identifier label on the needle. When a species was difficult to identify I used a similar label, with “vid. E. Geiser Year” to state that I have confirmed the identification. Specimens of the four species mentioned above were also studied carefully for the purpose of this revision. Here I did not tag my label to all of them, but mainly on vouchers that showed some variation of the typical characters or were collected in an interesting location.

The importance of museum collections has increased in recent years. Some areas of the Palaearctic region are inaccessible today for political or security reasons like Xinjiang in western China, or Syria, or Afghanistan. Fortunately, some natural history field studies dating more than 100 years ago enabled vouchers to be deposited in European museums. These historic specimens are an invaluable source for further studies. Additionally, it is more difficult today to collect Donaciinae and therefore *Plateumaris* species because man-made changes of limnic and wetland environments are contributing to the decline of habitats and, therefore, of species. Many previous habitats no longer exist and the only chance to study specimens from these districts is to examine the museum vouchers.

#### Main literature sources

In addition to the museum specimens studied, the author relied on certain important publications for additional data, mainly Hayashi (2020), Askevold (1991), and Bieńkowski (2014). Hayashi (2020) is a comprehensive recent study on Japanese Donaciinae which comprises five of the ten Palaearctic *Plateumaris* species occurring. Two species (*P.akiensis* and *P.constricticollis* with its three subspecies) are endemic in Japan, *P.shirahatai* is restricted to the East Palaearctic, and *P.sericea* and *P.weisei* occur across the Palaearctic region. Some figures and texts parts from Hayashi (2020) are cited here. The extensive study of Askevold (1991) on the Nearctic *Plateumaris* species is also important because it contains significant information about the Palaearctic *Plateumaris* species, and much information about the taxonomic aberrations affecting the genus. The comprehensive book of Bieńkowski (2014) on the Donaciinae of Russia was also helpful because this country covers a large part of the Palaearctic region and most of the Palaearctic *Plateumaris* species occur here. It also contains information about biology, ecology, food plants, and larvae of *Plateumaris*.

#### Original descriptions and type specimens

The study of original taxonomic descriptions is essential for any systematic revision. Many of the species or subspecies were first described in Latin with very specific phrases used in scientific entomological scripts in the 18^th^ and 19^th^ centuries. Some original descriptions are in German, French, or Russian; some are multilingual, starting with Latin for the main characters of a presumed new species, and then more details were added in the native language of the author. Therefore, to overcome these challenges, Geiser and Geiser (2023) present the original descriptions and their translations of all Palaearctic *Plateumaris* species including many synonyms. That comprises seven species, because three were originally described in English, and 19 taxa are now synonyms.

The study of type specimens is also essential in revisions, and I was able to examine six type specimens. Many types from the 18^th^ and 19^th^ centuries could not be located or no longer exist. Another problem is that specimens in collections may be labelled as “type” or indicated as types by red or red-bordered labels but are not actually type specimens.

#### Synonyms

For each species, in “Taxonomic history and synonymies” most synonymies are explained, where changes were made (Geiser in press) compared to Silfverberg (2010). Also, I attempted to provide the reference for the synonymisation. Although the literature was studied thoroughly, I could not track down this information in most cases. Some publications that are repeatedly cited in the literature in fact did not contain this information at all. Some synonyms listed here are confirmations of synonyms elaborated by previous authors but not accepted by some authors. In most cases new arguments are presented to validate these synonymisations.

### Distribution data

For each species, the countries, or the part of a country, with reliable records are listed. Detailed information on the data source is given for the countries or part of a country which are new in Geiser (in press) compared to Silfverberg (2010). The subdivisions of some large countries (e.g., Russia, China) are the same in this work as in Bezděk and Sekerka (in press).

## Results

### Identification keys

Many previous researchers have published keys to the Palaearctic species of *Plateumaris*, but these keys often use characters that are difficult to see from the beginning. Although only ten species occur in the Palaearctic region, some specimens are not easy to identify even for experienced coleopterologists. The new key reduces the species designation available for a particular specimen. The detailed characters and the pictures provided in the species sections should allow definitive identification. Because only two species occur in the whole Palaearctic region, the separation of the West and East Palaearctic in different keys leads to more reliable identifications. If the information on the specimen location label is very imprecise (e.g., “Siberia” or “Russia”) then both keys need to be applied.

### *Plateumaris* species which occur in the West Palaearctic

**Table:** 

1	Antennae and legs entirely metallic, same colour as pronotum and elytra. Sometimes the antennomeres can be reddish near the joints. At the legs small reddish parts may be occur near the joints or, exceptionally, on the tibiae or tarsomeres. Pronotum and elytra in various metallic colours (Fig. 11). Size: 6.5–10.5 mm. Occurs throughout the Palaearctic region	***P.sericea***
–	Antennae and legs yellow reddish brown, some parts more or less darkened	**2**
2	Pronotum and elytra black or with dark metallic lustre, elytra elongate, their length twice as long as wide, side contour of elytra parallel, not convex, the largest of all species (Fig. 4). Size: 8.0–12.0 mm. Occurs in most parts of the West Palaearctic except north Fennoscandia and southern Europe	***P.bracata***
–	Elytra not twice as long as wide	**3**
3	Pronotum cordate, upper side with metallic lustre in different colours or entirely black, elytra 1.5–1.8× longer than wide, never 2× longer than wide, side contour of elytra not parallel but slightly convex (Figs 4d, 5). Size: 6.0–9.2 mm. Occurs in most parts of the West Palaearctic, mainly in continental Europe including south Sweden, very rare in south and eastern Europe and west Siberia	***P.consimilis***
–	Pronotum not cordate	**4**
4	Pronotum distinctly flattened, almost quadrate, only slightly constricted at the basis, with flat disc and indistinct anterior tubercles, upper side bronze or black with greenish, bluish, or purplish metallic lustre, colour of pronotum and elytra mostly the same but can also differ significantly (Fig. 10). Size: 7.0–9.0 mm. Occurs in most parts of the West Palaearctic	***P.rustica***
–	Pronotum neither distinctly cordate nor flattened, outline subquadrate, slightly longer than wide, basal part narrowed, slightly cone-shaped, anterior tubercles distinctly visible or almost entirely smooth, upper side cupreous or bronze, sometimes metallic green, blue, purple, or non-metallic brown (Fig. 17; Table 3). Size: 6.2–8.0 mm. Trans-Palaearctic species, from northern Fennoscandia through Siberia to the Far East, northern China, the Korean peninsula, and Japan	***P.weisei***

**Remarks**: Some specimens of *P.weisei* are difficult to distinguish from *P.consimilis* and *P.rustica*. The variation of several characters is sometimes within the same range. It occurs also within the distribution area of *P.consimilis* and *P.rustica* in southeast Finland and some parts of Russia, though these three species are very rare here. To avoid misidentification, use all the details and figures provided in the species sections.

### *Plateumaris* species which occur in the East Palaearctic

**Table:** 

1	Femora and tibia entirely metallic in colour	**2**
–	Femora and tibia partly metallic or dark, femoral base usually rufous	**3**
2	Median line of pronotum deep, male pygidial apex usually emarginate; apex of median lobe of male genitalia without subapical corner; apex of median ejaculatory guide of endophallus rounded (Figs 11, 12a). Size: 6.5–10.5 mm. Occurs in the whole Palaearctic region	***P.sericea***
–	Median line of pronotum indistinct, male pygidial apex usually truncate; apex of median lobe of male genitalia with subapical corner; apex of median ejaculatory guide of endophallus notched (Figs 12b, 13). Size: 6.5–8.2 mm. Occurs in the East Palaearctic region	***P.shirahatai***
3	Metafemoral tooth present but blunt, pronotum neither distinctly cordate nor flattened, outline subquadrate, slightly longer than wide, basal part narrowed, slightly cone-shaped, anterior tubercles distinctly visible or almost entirely smooth, upper side cupreous or bronze, sometimes metallic green, blue, purple, or non-metallic brown (Fig. 17; Table 3). Size: 6.2–8.0 mm. Trans-Palaearctic species, from northern Fennoscandia through Siberia to Far East, northern China, the Korean Peninsula, and Japan	***P.weisei***
–	Metafemoral tooth well developed	**4**
4	Body robust in shape, with conspicuously short elytra and legs, elytra length is only 1.6× longer than width, at least always less than 1.7, pronotum finely and densely punctate; colour black or bronze-metallic, also with a bluish or greenish lustre, elytra shiny (Fig. 2; Table 3). Size: 6.4–7.6 mm. Endemic species of Honshu, Japan	***P.akiensis***
–	Elytra and legs not conspicuously short, normal, ratio of elytral length to width> 1.7	**5**
5	Pronotum cordate, surface shiny as if varnished, most parts without wrinkles or punctures, anterior tubercles protruding but smooth, gently narrowed with a shallow, transverse groove behind them, dorsal colouration variable (Fig. 7). Size: 6.6–11.9 mm. Occurs only in the Japanese Archipelago (Distinguishing characters of the three subspecies: 5a *P.constricticollisconstricticollis* – Colouration of pronotum and elytra differs between specimens. Femora and tibia entirely rufous, sometimes partly dark rufous, apex of cap of tegmen deeply and sharply notched in most specimens. 5b *P.constricticollisbabai* – Same colouration of pronotum and elytra, apical half of femora black metallic, tibia usually rufous, sometimes partly dark rufous, apex of cap of tegmen not deeply notched. 5c *P.constricticollistoyamensis* – Pronotum and elytra entirely metallic, apical half of femur black, sometimes darkly rufous, tibia usually rufous, sometimes partly dark rufous, apex of cap of tegmen slightly notched or rounded, subapical angle of ovipositor nearly right, apex slightly prominent, finely serrated subapically)	***P.constricticollis***
–	Pronotum not cordate, covered with rugae and punctures	**6**
6	Antennae and legs mostly rufous, sometimes apically darkened, femora reddish on the basal half and metallic-dark on the apical half (Fig. 9). Size: 6.7–9.7 mm. East of Lake Baikal to Far East, Sakha (Yakutia) Republic, Amur region in Russia, and northeast China	***P.roscida***
–	Legs usually rufous, sometimes apical area of femora dark (Fig. 3). Size: 7.1–7.7 mm. In Russia east of Lake Baikal, Sakhalin, and Kurile Islands	***P.amurensis***

**Remarks**: The three species found in the east of Russia and northeast China, *P.amurensis*, *P.roscida*, and *P.weisei*, are not easy to distinguish because their external characters sometimes overlap. To distinguish *P.amurensis* from *P.weisei* see also Table 3. The best distinguishing character of *P.roscida* is the dark colour on the apical half of the femora; especially on the metafemur where it appears that the femur had been dipped to the half-way point in a pot with brown paint. Unfortunately, there are some *P.roscida* specimens with entirely rufous legs. The best differential character is then the aedeagus, which is very different from those of all the other Palaearctic *Plateumaris* species: the apex of the median lobe shows a conspicuous elongated peak and the cap of the tegmen has a deep, narrow apical notch (Fig. 9).

#### Opinions on species delimitation in the literature

Although only a few Palaearctic *Plateumaris* species exist (compared with other beetle genera), there are many differing opinions between authors. Almost every comprehensive publication about Palaearctic *Plateumaris* shows a different number of species (Table 1).

The highest number of species is recorded by Borowiec (1984): he regarded 17 species as valid. At that time *P.akiensis* was not described and *P.amurensis* was regarded as a synonym of *P.weisei*. Therefore, two more species can be added. Goecke (1960) mentioned 13 *Plateumaris* species for the Palaearctic region in his world checklist; three were not described then and he regarded *P.amurensis* as a synonym. Therefore, four more species must be added. In his major work about the Nearctic *Plateumaris* species, Askevold (1991) also treated the Palaearctic species. He established several synonyms, some of them as probable new synonyms. He regarded nine names as valid for Palaearctic *Plateumaris* species. I agree with his assessment except concerning *P.amurensis*, which he regarded to be synonymous with *P.weisei*. Hayashi (2001) showed that *P.amurensis* is a separate species, bringing the total to ten species.

Two major books were published in 2010: Warchalowski treated 12 species in his identification key and Silfverberg listed 16 species (and some more subspecies) in Löbl and Smetana (2010). Some large works which cover only a part of the Palaearctic region also contain invaluable information such as Bieńkowski (2014) on the Russian Donaciinae, which covers a large part of the Palaearctic region and almost all the Palaearctic *Plateumaris* species. The recent publication by Hayashi (2020) deals with the five Japanese *Plateumaris* species, which includes half of all the Palaearctic species; the paper contains the species that do not occur in Russia, so the Palaearctic region is completely covered with these two publications. Gressitt and Kimoto (1961) named four *Plateumaris* species in their comprehensive study on the Chrysomelidae of China and Korea; except for *P.amurensis* the other three names are synonyms.

**Table 1.** Comparison of major publications: names of Palaearctic species of the genus *Plateumaris* Thomson including the different opinions about valid species and synonyms.

*Plateumaris* species names	Palaearctic Region					Parts of Palaearctic Region		
	Silfverberg 2010	Warchalowski 2010	Askevold 1991	Borowiec 1984	Goecke 1960	Japan: Hayashi 2020	Russia: Bienkowski 2014	China & Korea: Gressitt and Kimoto 1961
*P.affinis*	*	syn of *P.rustica*	syn of *P.rustica*	*	*	—	syn: *P.rustica*	—
*P.akiensis*	*	*	*	o: described in 1984	o: described in 1984	*	—	—
*P.amurensis*	*	*	“syn of *weisei* by Goecke which is probably correct”	o: clarified 2001 to be different from *P.weisei*	syn of *P.weisei*	—	*	*
*P.bracata*	*	*	*	*	*	—	*	o: first record 2023
*P.caucasica*	*	o	possible syn of *P.roscida*	*	o	—	ssp of *P.sericea*	—
*P.consimilis*	*	*	*	*	*	—	*	—
*P.constricticollis*	* including 3 ssp	*	*	*	*	* including 3 ssp.	—	—
*P.discolor*	*	syn of *P.sericea*	syn of *P.sericea*	*	*	syn of *P.sericea*	*	—
*P.mongolica*	syn of *P.weisei*	syn of *P.weisei*	probable syn of *P.weisei*	*	*	syn of *P.weisei*	—	*
*P.obsoleta*	*	*	probable syn of *P.sericea*	*	*	—	*	—
*P.roscida*	*	*	*	*	*	—	*	as *P.annularis*
*P.rustica*	*	*	*	*	*	—	*	—
*P.sachalinensis*	syn of *P.weisei*	*	probable syn of *P.weisei*	*	o: described in 1973	syn of *P.weisei*	*	—
*P.sericea*	*	*	*	*	*	*	*	as *P.socia*
*P.shirahatai*	*	as syn of *P.obsoleta*	*	*	o: described in 1971	*	*	o: described in 1971
*P.sibirica*	ssp of *P.sericea*	ssp of *P.sericea*	o	o	syn of *P.sericea*	ssp of *P.sericea*	ssp of *P.sericea*	—
*P.socia*	syn of *P.sericeasibirica*	syn of *P.sericeasibirica*	probable syn of *P.sericea*	*	o	syn of *P.sericea*	—	*
*P.sulcifrons*	*	*	probable syn of *P.rustica*	*	*	—	—	—
*P.tenuicornis*	*	o	probable syn of *P.consimilis*	*	*	—	—	—
*P.weisei*	*	*	*	*	*	*	*	o
**Number of valid species**	**16**	**12**	**9**	**17 (+2)**	**13 (+4)**			

* = valid species; o = not mentioned although it occurs in the studied area; — = does not occur in the studied area; ssp = subspecies; syn = synonym.

### Taxonomic accounts

#### Genus Plateumaris

Taxon classificationAnimaliaColeopteraChrysomelidae

C. G. Thomson, 1859

D67081AF-379C-5CF8-AC9A-5EC462C7DD87

Plateumaris C. G. Thomson, 1859: 154.
Euplateumaris Iablokoff-Khnzorian, 1966: 121.
Juliusina Reitter, 1920: 41.

##### Type species and localities.

*Plateumaris* C. G. Thomson, 1859: *Donacianigra* Fabricius, 1792: Germania.

= *Prionusbracatus* Scopoli, 1772: Carniola [now Slovenia].

*Euplateumaris* Iablokoff-Khnzorian, 1966: *Lepturasericea* Linnaeus, 1758: Europe.

*Juliusina* Reitter, 1920: *Prionusbracatus* Scopoli, 1772: Carniola [now Slovenia].

##### Timeline of taxonomic history and synonymies.

1758: Linnaeus described the genus *Leptura* with 22 species. Two of these species belong to the (later established) Donaciinae: *Donaciaaquatica* and *Plateumarissericea* (Geiser and Geiser 2023). Linnaeus established only the taxonomic categories: classis – Coleoptera – genus – species, e.g.: Insecta – Coleoptera – Leptura – sericea.

1760: Linnaeus mentioned *Lepturasericea* in his ‘Fauna Suecica’ with the same diagnosis as in Linnaeus (1758) but with more details. Therefore, the species is sometimes cited as *Lepturasericea* Linnaeus, 1760 (e.g., in Silfverberg 2010), but the first description date is clearly Linnaeus, 1758 (see Geiser and Geiser 2023 for original Latin text of Linnaeus and translation).

1762: Geoffroy erected the genus *Prionus* for the species *Cerambyxcoriarius* Linnaeus, 1758. It now belongs to the Cerambycidae as does the genus *Leptura*.

1772: Scopoli described *Prionusbracata*.

1775: The genus name *Donacia* was erected by Fabricius. He described *Donaciacrassipes* and *Donaciasimplex* and assigned *Lepturaaquatica* L., 1758 to the genus *Donacia*, but he did not change the genus name of *Lepturasericea* L., 1758. Other *Plateumaris* species were described as *Leptura*, e.g., *Lepturaconsimilis* Schrank, 1781. It is remarkable that these early entomologists had already assigned them to a genus other than *Donacia*.

1796: The category “Chrysomelidae” was established between order and genus for insects by Latreille.

1802: Latreille established the coleopteran family Chrysomelidae.

1837: Kirby established the subfamily Donaciinae.

1859: The genus name *Plateumaris* was erected by C. G. Thomson (translation in Geiser and Geiser 2023), but some authors, especially Americans, preferred *Donacia* as the genus name in their new descriptions. Even Marx (1957) regarded *Plateumaris* as a subgenus of *Donacia*. The Palaearctic *Plateumarisweisei* (Duvivier, 1885) and *P.constricticollis* (Jacoby, 1885) were also originally described as *Donacia*.

1920: Reitter split the Palaearctic *Plateumaris* into two subgenera: *Plateumaris* sensu stricto and *Juliusina*. He assigned *P.sericea* and *P.discolor* to *Plateumaris* s. str. and he described *P.annularis* in a footnote to *P.sericea*. In the same footnote he assigned *P.obsoleta* to *P.annularis*, but not as a synonym, and he placed *P.amurensis* near to *P.discolor*. The subgenus Juliusina contained *P.bracata*, *P.consimilis*, *P.rustica*, and *P.affinis*. To the latter species he assigned *P.sulcifrons* and *P.mongolica*. Moreover, Reitter made no designation of type specimens to either of the new subgenera.

##### Type species designations.

Thomson (1859) stated “Typus *P.nigra* (FAB.) [= Fabricius]” in the original description of the genus *Plateumaris*, but most authors cited “Thomson, 1866” as the year of the original description. Thomson (1866) is an extensive book but contains no type designation because this had been done previously in 1859. Therefore, the prevailing opinion was that there existed no type designation for the genus *Plateumaris* (which was wrong) and nor for the subgenera *Juliusina* and *Plateumaris* s. str (which is true). Subsequently, Chen (1941) designated *Donaciaaffinis* Kunze as the type species for *Plateumaris*, but this was overlooked by Monrós (1959) who designated *Donaciageniculata* Thomson (= *Donaciadiscolor* Panzer) as the type species for *Plateumaris* s. str. and *Prionusbracatus* Scopoli for *Juliusina* Reitter.

Iablokoff-Khnzorian (1966) suggested the name Euplateumaris for the subgenus Plateumaris s. str. established by Reitter, with *Donaciasericea* (L.) as type species. He also re-established *D.nigra* F. (= *bracata*) as type species of the genus *Plateumaris*, apparently unaware of both designations by Monrós (1959) and Chen (1941), and therefore placed *Juliusina* as a junior synonym of *Plateumaris* because both were named based on the same nominal taxon. Jolivet (1970) followed the designation of Iablokoff-Khnzorian (1966) but Mann and Crowson (1983) accepted Monrós’ designations and advocated acceptance of *Plateumaris* s. str (= *Euplateumaris*) and Plateumaris (Juliusina) as the correct subgeneric classifications of *Plateumaris*. Lopatin (1984) correctly cited *P.bracata* as type species of *Plateumaris* but he assigned this species to Plateumaris (Juliusina). The same, clearly incorrect, arrangement was used by Lopatin and Kulenova (1986) when assigning *P.sericea* to Plateumaris (Plateumaris).

The names *Plateumaris* Thomson, 1859 and *Juliusina* Reitter, 1920 are based on the same type species (*Prionusbracatus*) which makes the subgenus Juliusina a synonym of *Plateumaris* in the sense of the whole genus, which was endorsed by Warchałowski (2010). This synopsis of the type species designations and the use of the names as described was elaborated in detail by Askevold (1991). Also, he was the first who attempted to assess the validity of *Plateumaris* as a genus and the validity of the two subgenera.

##### Taxonomic status of *Plateumaris*.

Most European authors accepted the genus *Plateumaris*, but North American authors were reluctant and oscillated between the use of this name at generic and subgeneric ranks within the genus *Donacia*. This ambiguity led to confusion about the genus name *Plateumaris*, worsening the existing confusion over the name *Plateumaris* of the Palaearctic species (see above).

Askevold (1990) understood that *Plateumaris* was monophyletic, defined by the synapomorphy of the ovipositor structure, and that *Plateumaris* was the sister group to all other Donaciinae, and his morphologically based conclusions were confirmed by several independent molecular analyses (Sota and Hayashi 2007; Kölsch and Pedersen 2008; Sota et al. 2008; Hayashi and Sota 2014; Reis et al. 2020).

##### Previous subgeneric classifications.

Askevold (1990, 1991) concluded that the division into two subgenera by Reitter (1920) did not reflect the phylogenetic reality. The two subgenera were erected based on characters that do not occur in all members assigned to them or they were based on plesiomorphic characters. Notably, neither subgenus can be characterised by a synapomorphy. His conclusion that *Euplateumaris* and *Juliusina* cannot be regarded as subgenera has also been confirmed by molecular studies (Kölsch and Pedersen 2008; Sota et al. 2008). Therefore, this paper does not deal with the confusion over subgeneric names and various designated type species.

In central Europe, the widely used key of Mohr (1966a) separated *Plateumaris* into two subgenera. The short note in the updated key by Kippenberg (1994: 22), “The separation into the subgenera *Juliusina* Rtt. and *Plateumaris* s. str. is not tenable,” did not prevent coleopterologists from using the subgeneric names in their collections or publications, and even on a Palaearctic scale, the subgeneric names *Euplateumaris* and *Juliusina* were still used by Silfverberg (2010) and Bieńkowski (2014). Possibly the title ‘Classification, Reconstructed Phylogeny, and Geographic History of the New World Members of *Plateumaris* Thomson, 1859’ of Askevold (1991) suggested that this publication dealt only with new world *Plateumaris* species and did not contain relevant information on Palaearctic *Plateumaris* species or their subgeneric statuses.

##### Diagnosis of the genus *Plateumaris* Thomson, 1959.

**Figure 1.**
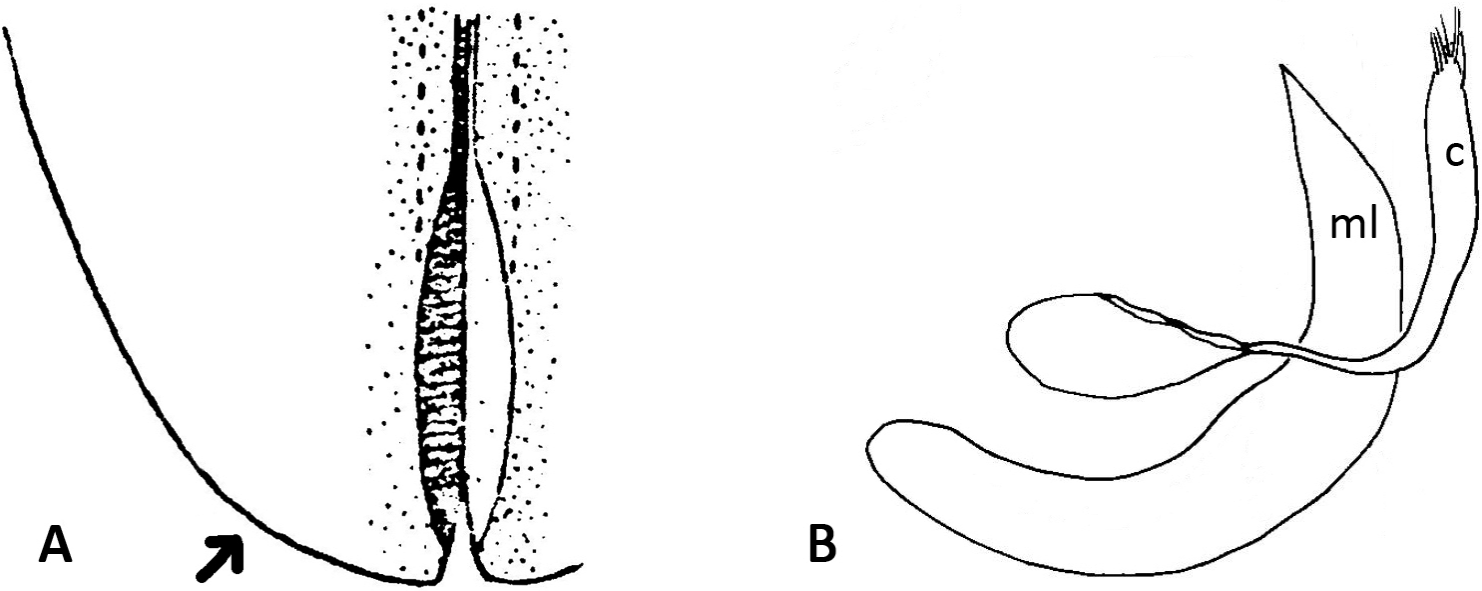
Diagnostic characteristics of *Plateumaris* sp. **A** elytral apex with sutural margin broadly exposed, no outer angle protruding (arrowed; from Kaszab 1962) **B** typical structure of aedeagus in lateral view (from Askevold 1991). Abbreviations: **ml** median lobe, **c** cap of tegmen.

Askevold (1991) detailed the diagnosis so only the most necessary characters needed to distinguish the *Plateumaris* species from all other Donaciinae are listed below:

- Sutural margin of elytron explanate apically, sutural interval sinuates distinctly before apex, lower sutural margin broadly exposed (Fig. 1A);
- Elytral apex rounded, inner angle sharp, no outer angle protruding;
- First abdominal segment as long as the others combined;
- Host plants are typically Cyperaceae, but also a few other wetland plants.

The aedeagus of *Plateumaris* species and some other representatives of the Donaciinae consists of a median lobe which contains the endophallus and the lateral parameres (Fig. 1B). The parameres are fused basally and distally, forming a ring around the median lobe. This parameric structure is the tegmen, composed of a ventral strut and a dorsal cap (for more details and functional descriptions see Askevold 1991 and Hayashi 2020). The frontal view towards the apex of the median lobe and of the cap of tegmen are usually very characteristic of each *Plateumaris* species and therefore suitable to distinguish them in most cases. Some species can be distinguished only by subtle morphological characters of their endophallus.

##### Biology.

Reed beetles live on plants in wetlands. The larvae develop attached to the roots in the sediment and live as sap suckers gnawing a hole into the root. They breathe by tapping the aerenchyma of the plant using two hollow abdominal hooks, which are connected to their tracheal system; therefore, they can live even in anoxic mud (Böving 1910). Adults remain mostly above the surface sitting on aquatic plants but are able to hide under water while the ventral hairs serve as plastron respiration (Rheinheimer and Hassler 2018). The larvae pupate at the end of their second summer in a cocoon, attached to the root of the food plant. The beetle overwinters in an air-filled cocoon and emerges the following spring. In warmer climates the larvae may pupate after their first summer (Böving 1910; Kleinschmidt and Kölsch 2011).

The larval host plants are mostly members of Cyperaceae, but include some Juncaceae and Poaceae (e.g., *Phragmitesaustralis* (Cav.) Trin. ex Steud.). *Acoruscalamus* L. (Araceae), *Calthapalustris* L. (Ranunculaceae), and *lris versicolor* L. (Iridaceae) are also mentioned as food plants (Borowiec 1984; Bieńkowski 2014). Most species are mono- or oligophagous, especially the larvae. Adult beetles feed on the leaves, although some species are pollen feeders, and some may also feed on petals. If a larva is attached to the root of a plant and gnawing traces are found, we can be sure that this is a host plant for this species. Adults may use a slightly broader range of plants. Several species feeding on pollen use the plants of their habitat. Therefore, adults can be observed on wetland plants that are mostly not larval food plants. The adults are active mainly in spring and early summer, sometimes also in autumn. No specific phenology data are provided here for the species because many have a wide distribution range and the time of “spring” differs between the populations within the Palearctic region.

*Plateumaris* species prefer habitats of wet sedge meadows, peat bogs, and fens, in contrast to *Donacia* species which live on aquatic plants with emerging parts and in contrast to *Macroplea* species which live on totally submerged aquatic plants.

##### Historical biogeography.

*Plateumaris* species are found only in the Holarctic region with ten species in the Palaearctic and 17 species in the Nearctic (Askevold 1991); the latter occur mainly in Canada and the northern states of the USA. These two subregions have neither a *Plateumaris* species nor any other Donaciinae species in common, although molecular analysis shows that many Palaearctic *Plateumaris* species are more closely related to Nearctic species than to other Palaearctic species (Askevold 1991; Hayashi 2020). Dispersal vicariance analysis and divergence time estimation revealed that the European and North American-Asian lineages diverged during the Eocene. Moreover, subsequent differentiation occurred repeatedly between North American and Asian species, which was facilitated by three dispersal events from North America to Asia and one in the opposite direction during the late Eocene through the late Miocene (Kölsch and Pedersen 2008; Sota et al. 2008; Hayashi and Sota 2014).

##### Checklist and distribution.

A summary of the distribution of the *Plateumaris* species in the Palaearctic region is shown in Table 2.

**Table 2.** Checklist and distribution of the *Plateumaris* species in the Palaearctic region.

*Plateumaris* species		Distribution
1	*P.akiensis* Tominaga & Katsura, 1984	Japanese endemic: records only from Hiroshima prefecture so far
2	*P.amurensis* Weise, 1898	Russia: East Siberia and Far East
3	*P.bracata* (Scopoli, 1772)	West Palaearctic except of south Mediterranean including Kazakhstan and west Siberia
4	*P.consimilis* (Schrank, 1781)	Europa and west Siberia
5	*P.constricticollis* (Jacoby, 1885)	Endemic of the Japanese archipelago
6	*P.roscida* Weise, 1912	East Siberia and Far East, from northern China to Amur River region, Lake Baikal, and the Sakha (Yakutia) Republic
7	*P.rustica* (Kunze, 1818)	West Palaearctic
8	*P.sericea* (Linnaeus, 1758)	Palaearctic
9	*P.shirahatai* Kimoto, 1971	East Palaearctic: Russian Far East, Japan, and South Korea
10	*P.weisei* (Duvivier, 1885)	Northern Europe and northern Asia, Japan, and South Korea

**Palaearctic region**: *Plateumarissericea* has the largest distribution area of any Donaciinae species: it is recorded from Ireland and Great Britain to the whole of continental Europe, North Africa, and almost all Asian countries which belong to the Palaearctic region. *Plateumarisweisei* occurs from northern Europe to east Asia, from Siberia to northern China, Japan (Hokkaido), and South Korea.

**Western Palaearctic region**: Three species occur only in Europe and in Asia west of Lake Baikal: *P.bracata*, *P.consimilis*, and *P.rustica*.

**Eastern Palaearctic region**: Five species occur only in Asia east of Lake Baikal: *P.amurensis*, *P.roscida*, and *P.shirahatai*, and two species are endemic to the Japanese archipelago, *P.akiensis* and *P.constricticollis*.

#### Palaearctic species of the genus *Plateumaris*

##### Plateumaris akiensis

Taxon classificationAnimaliaColeopteraChrysomelidae

Tominaga & Katsura, 1984

FB0093D9-D5C9-5BCE-9F4B-E01A29FF661B

Fig. 2

Plateumaris akiensis Tominaga & Katsura, 1984: 25.

###### Type locality.

Japan, Honshu, Shinkawa-tameike, altitude 770 m, Nishi-yawata-hara, Geihoku-cho, Yamagata-gun, Hiroshima Prefecture.

###### Type material.

***Holotype***: Japan • ♂; Honshu, Hiroshima Prefecture, Yamagata-gun, Geihoku-cho, Nishi-yawata-hara, Shinkawa-tameike; 770 m a.s.l.; 13 Jun 1982; I. Hiura leg.; OMNH-TI-16. The holotype was not examined.

###### Taxonomic history.

Because this species was not described until 1984 it is possible that some small *Plateumaris* specimens from Japan are misidentified as another species in old collections.

###### Diagnosis.

**Figure 2.**
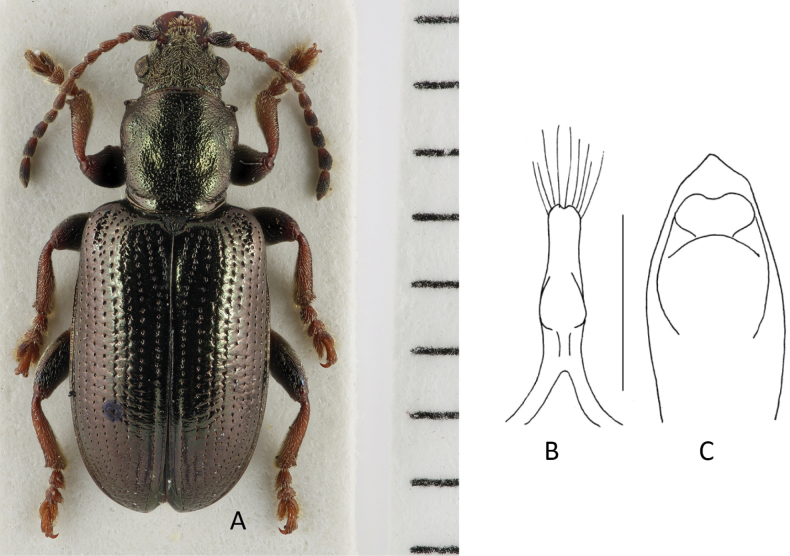
*Plateumarisakiensis***A** habitus (photograph by K. Matsumoto) **B** cap of tegmen **C** median lobe (**B, C** from Hayashi 2020). Scale bars: one unit – 1 mm (**A**); 0.5 mm (**B, C**).

Habitus (Fig. 2A) like a typical *Plateumaris*, but with conspicuously short elytra and legs, elytra length is at the most 1.6× longer than wide, colour black or bronze-metallic, also with a bluish or greenish lustre, elytra shiny, antennomeres thicker than in most *Plateumaris* species, legs reddish but femora dark.

###### Description.

There are comprehensive descriptions in Tominaga and Katsura (1984) and in Hayashi (2020) with many detailed figures so the description below is confined to the essentials.

***Size***: 6.4–7.6 mm.

***Colour***: Colour of pronotum and elytra entirely metallic black or reddish coppery, some specimens with a bluish or greenish lustre.

***Head***: Supraocular furrow indistinct, frontal tubercle weakly convex.

***Antennae***: Antennae short and robust, slightly shorter than half as long as the body, A1 dark, A2–A11 entirely reddish brown or apical antennomeres with darker parts. A1 is longest, A2 is shortest, A3–A11 are only slightly longer than A2, A3 ≥ A4 ≤ A5.

***Pronotum***: Slightly longer than wide, outline more or less cordate, gradually narrowed backwards with anterior margin strongly produced forward; dorsal surface with feebly raised anterior and posterior tubercles, disc densely but finely punctate, median line shallow, with a conspicuous broad collar along the posterior margin.

***Elytra***: Robust and short, 1.6× as long as wide, with arched outer margin, rows of punctures regular, their interstices smooth, usually sparsely but sometimes densely punctate.

***Legs***: Legs short, reddish brown, partly dark rufous or black, especially the apical half of the femora, metafemur robust in shape, with a tooth; T2 shorter than its width, T1 > T2 < T3.

***Pygidium***: Entirely rufous, but apical part black, apex pubescent, shallowly emarginate in both sexes.

***Aedeagus***: See Fig. 2B, C.

###### Similar species.

*Plateumarisakiensis* looks similar to *P.constricticollis* but can be identified by its shorter elytra. In *P.constricticollis* the elytral length is distinctly > 1.6× longer than wide.

###### Biology.

The adults feed on pollen of *Carex* sp. and *Scirpusjuncoides* (Hayashi 2020). Narita (2003) described the larvae from *Carexotaruensis* Franch. *Scirpus* sp. is also thought to be a larval host plant (Hayashi 2020).

###### Distribution.

Endemic species found in Honshu, Japan. The only records so far are from Hiroshima and Shimane prefectures in south-west Honshu.

###### Material examined.

Five specimens from Hiroshima prefecture, stored in BMNH, HNHM, and NMPC.

##### Plateumaris amurensis

Taxon classificationAnimaliaColeopteraChrysomelidae

Weise, 1898

FD3326FA-F15B-5A96-919E-EA9B39A1AAD8

Fig. 3

Plateumaris amurensis Weise, 1898: 179.

###### Type locality.

Amur [assumed Russia, further details unknown].

###### Type material.

Types could not be located so far. In his original description (translation in Geiser and Geiser 2023), Weise gave no indication from whom he received the material, where the type is stored, nor the number of specimens he studied.

**Figure 3.**
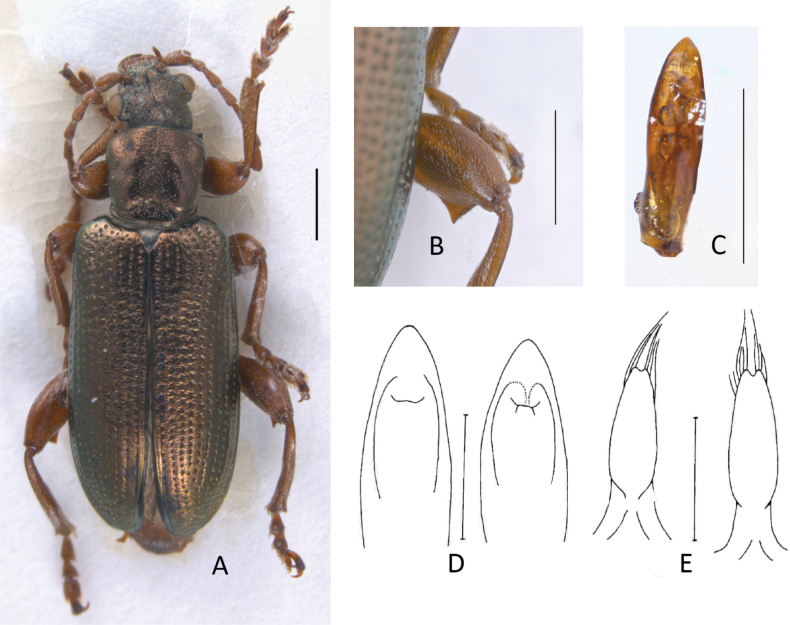
*Plateumarisamurensis***A** habitus **B** metafemur with prominent tooth **C** aedeagus (**A–C** photographs by E. Geiser) **D** median lobe **E** cap of tegmen (**D, E** from Hayashi 2001). Scale bars: 1 mm (**A–C**); 0.5 mm (**D, E**).

###### Taxonomic history and synonymies.

Weise (1898) assumed a relationship to *P.discolor* and further compared it with *P.weisei* that is similar in colour. Reitter (1920) mentioned both *P.amurensis* and *P.weisei* in a footnote with some characters from their original descriptions but not in his identification key. He also noted no distinguishing characters. Both species are also mentioned in the catalogue of Winkler (1930). Goecke (1937) regarded *P.amurensis* as synonymous with *P.weisei* in his modification to Reitter’s (1920) identification key, but he provided no reason or reference for this synonymy and subsequently, *P.amurensis* was considered a synonym (Goecke 1960; Jolivet 1970). Askevold (1991) agreed with Goecke’s view. Only in the key of Gressitt and Kimoto (1961) is *P.amurensis* regarded as a species propria with different characters to *P.mongolica* (which is now a synonym of *P.weisei*) but those characters are not adequate to distinguish these two species. Medvedev (1992) regarded *P.amurensis* a valid species; Hayashi (2001) then confirmed that *P.amurensis* was a species propria. Many external characters are highly variable and sometimes overlap with the characters of *P.weisei*. Due to the situation described above, specimens of *P.amurensis* are sometimes hidden in collections because they were identified as *P.weisei*.

###### Diagnosis.

Metafemur with a prominent, blade-like tooth, apical part of median lobe of aedeagus gradually narrowed towards the apex.

###### Description.

***Size***: 7.1–7.7 mm.

***Colour***: Pronotum and elytra bronze, cupreous, also which greenish reflex.

***Head***: Supraocular furrow indistinct, vertex pubescent with deep median line. Antennae entirely rufous, sometimes apex darkly rufous; A4 = 1.6× A2, A5 longest and ~ 2.5× as long as wide.

***Pronotum***: More or less quadrate, anterior part slightly widened by shallow anterior tubercles, disc shiny, coarsely punctate, rugose, sometimes with microsculpture in major part of the disc, basal sulcus prominent with rugae and dense punctures, median groove indistinct.

***Elytra***: Sparsely rugose, shiny, densely punctate on disc.

***Legs***: In most specimens rufous, sometimes apical area of femora dark, metafemur with a prominent, blade-like tooth (Fig. 3B; Table 3).

***Pygidium***: Apex pubescent, shallowly emarginate or sometimes truncate in male and rounded in female. Last sternite entirely coppery but apex at middle part rufous, apical shape variable in male, acute in female (Table 3).

***Ovipositor***: Elongate, both sides paralleled, apical angle acute, subapical corner with teeth, apex remarkably prominent (Table 3).

***Aedeagus***: With median lobe, acute but slightly rounded at the apex; cap of tegmen gradually narrowed distally, notched, or sometimes rounded at apex (Fig. 3C–E).

###### Similar species.

The most similar species is *Plateumarisweisei* and the main distinguishing characters are shown in Table 3. Otherwise, the East Palaearctic *Plateumaris* species are not easy to distinguish. I found specimens of *P.amurensis* in museums also identified as *P.roscida*, *P.sericea*, or as their synonyms.

###### Biology.

Host plant and larvae are unknown.

###### Distribution.

East Palaearctic only. Records exist for Asia: Russia: Transbaikalia, Republic of Sakha (Yakutia): southern part of river Lena; Amur Oblast, Khabarovsk Krai, Primorsky Krai, Sakhalin, Kurile Islands. Sometimes *P.amurensis* is mentioned from Japan (e.g., Bieńkowski 2014) but these are erroneous records caused by confusion with *P.weisei* (Hayashi 2001, 2020).

###### Material examined.

15 specimens from different localities in East Siberia and Far East.

**Table 3.** Distinguishing characters of *Plateumarisamurensis* und *P.weisei* (drawings from Hayashi 2001).

	*P.amurensis*		*P.weisei*	
Metafemur	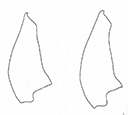		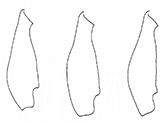	
Last sternite male	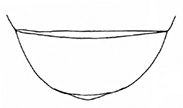		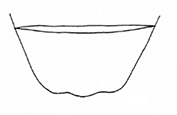	
Last sternite female	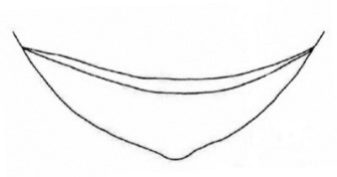		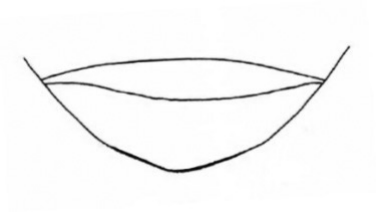	
Aedeagus: apex of median lobe	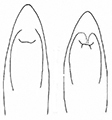		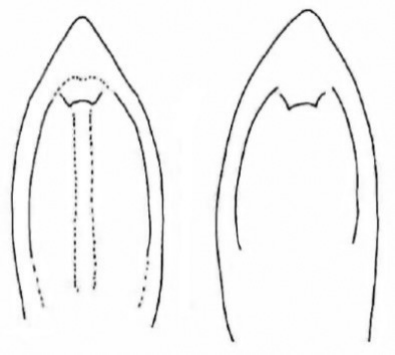	
Cap of tegmen	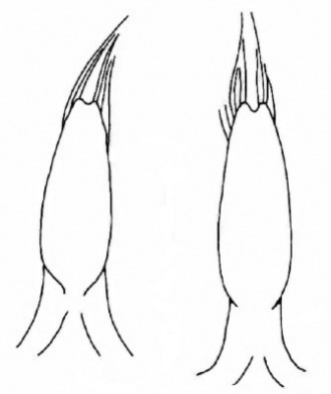		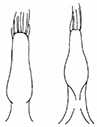	
Ovipositor: ventral view and apex contour	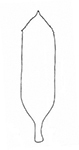	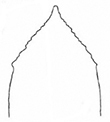	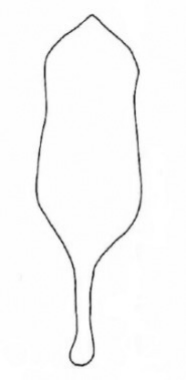	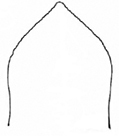

##### Plateumaris bracata

Taxon classificationAnimaliaColeopteraChrysomelidae

(Scopoli, 1772)

986E9B52-3535-5199-BB29-A58FF249F8BF

Fig. 4

Prionus bracatus Scopoli, 1772: 100.
Donacia abdominalis Olivier, 1800: 9.
Plateumaris bracatus var. fairmairi LeGrand, 1861a: 265.
Donacia nigra Fabricius, 1792: 117.
Donacia palustris Herbst, 1784: 100.
Leptura violacea Pallas, 1773: 724.

###### Type locality.

*Plateumarisbracatus*: Carniola, a historical region which comprised parts of present-day Slovenia.

###### Type material.

Type specimens of *P.bracatus* do not exist anymore. Sadly, Scopoli’s collection of insects from Carniola decayed during his life time. He committed this collection to a printer in Vienna who apparently did not store it adequately. Before all the pictures were printed, Scopoli complained in his 1773 letter to Linnaeus (Brelih et al. 2006) that the insects “had either decayed or fallen apart”.

**Figure 4.**
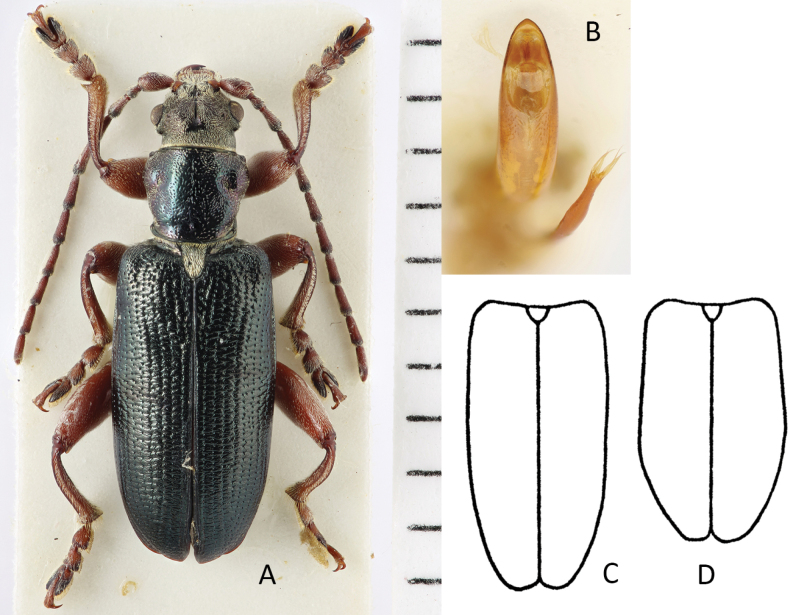
*Plateumarisbracata***A** habitus **B** aedeagus (photographs by K. Matsumoto) **C** elytra **D** elytra of *Plateumarisconsimilis* (drawings from Mohr 1966a). Scale bar: one unit – 1 mm.

###### Taxonomic history and synonymies.

In nearly all publications the species name has been misspelled “braccata”. The spelling in the original description by Scopoli (1772) is “Prionus Bracatus” with a single “c” (Geiser and Geiser 2023). Scopoli used this epithet presumably because “bracae” means “trousers” or “pants” referring to the clubbed shape of the metafemora. A variant of this word exists in later medieval Latin and was spelled “braccae”, which Schenkling (1917) used in his explanation of the scientific names of beetles. Because the spelling of the original description is linguistically correct it must be preserved unaltered (ICZN, Art. 32.2.1). Because the original description by Scopoli is very short, Weise (1893) published a more detailed redescription (see Geiser and Geiser 2023).

*Donaciaabdominalis* (Olivier, 1800): Silfverberg (2010) cited this synonym as *abdominalis* (Olivier, 1795). Despite the title page dated 1795, the fourth volume of Olivier’s ’Entomologie, ou histoire naturelle des insectes’ was issued in two parts, one probably in 1795 and the second one in 1800. All new taxa made available in this work have previously been dated 1795 in the literature (Bousquet 2018). In any case, Olivier’s volume does not contain the original description. He also listed another name of this species, *Lepturaviolacea* Pallas, and then described the typical characters of *P.bracata*. Later, *P.abdominalis* was regarded synonymous with *P.rustica* because it was mixed up with *P.abdominalis* Bedel, 1891, who intentionally did not describe it as a new species (see section on *P.rustica* for details).

*Plateumarisfairmairi* was first described by LeGrand (1861a) on page 265 as variation of *Donacianigra* (see Geiser and Geiser 2023) from a specimen with complete black antennae and legs. The often-mentioned page 89 derives from the reprint (LeGrand 1861b).

Fabricius described *Donacianigra* by 1792 (see Geiser and Geiser 2023). He allocated it close to *D.festucae* which is now synonymous with *P.sericea*. The name *Plateumaris* was not established then, but Fabricius (1792) noticed some differences from other *Donacia* species.

###### Diagnosis.

This is the largest *Plateumaris* species, body length: 8.0–12.0 mm (Fig. 4A).

Head, pronotum and elytra entirely black or with a weak blue, purple or green metallic lustre, antennae and legs reddish brown in most specimens, elytra elongate, ~ 2× as long as wide, side contour of the elytra very straight, almost parallel.

###### Description.

***Size***: 8.0–12.0 mm.

***Colour***: Dark, entirely black or at the most with weak blue, purple or green metallic lustre.

***Head***: Entirely black or with weak metallic lustre. Fine punctures and very fine wrinkles. Antennae minimum as long as the half length of the beetle, basal part of the antennomere always reddish (with rare exceptions), apical part dark; ½ A1 ≥ A2, ½ A3 ≥ A2, A4 ≥ A3, A1 ≈ A4, A5 … A11 ≈ A4.

***Pronotum***: Cordate, ahead distinctly wider than behind, anterior tubercles only slightly convex, slightly pubescent. Disc with fine punctures, median line well developed, sometimes shortened, but also very shallow and almost invisible in some specimens.

***Elytra***: Base of elytra with short, distinct setae in most specimens, elytral disc rugose, punctures delicate, not deep, interstices strongly transversely wrinkled; contour of the margin very straight, almost parallel; elytra elongate, ~ 2× as long as wide, ratio of length to width = (1.8–2.1): 1.

***Legs***: Colour variation from complete reddish brown to dark apical parts and completely dark legs, femora basally very broad, metafemur with broad tooth, robust in male, in female feeble or indistinct.

***Aedeagus***: see Fig. 4B.

###### Similar species.

The most similar species is *Plateumarisconsimilis*, which is smaller (6.0–9.2 mm) on average, its elytra are shorter with a ratio of length to width ≤ 1.8, and the outer contour of the elytra are slightly rounded, not parallel.

###### Biology.

*Plateumarisbracata* is monophagous on *Phragmitesaustralis* (Cav.) Trin. ex Steud., the common reed, Poaceae (Bieńkowski 2014). The beetle can be found concealed in the *Phragmites* leaf-folds. When feeding, it penetrates the young leaf shoots which later unfold to present a characteristic transverse series of round holes. *Donaciaclavipes* feeds on the same plant species in a similar fashion but, in this case, the series of holes produced are irregularly elongated (Menzies and Cox 1996; Rheinheimer and Hassler 2018). For identification of the larvae see Steinhausen (1994) and Bieńkowski and Orlova-Bieńkowskaja (2004). Despite its large distribution area and its common food plant, its number of specimens stored in museum collections is always remarkably fewer than the number of *P.consimilis* or *P.sericea*. Compared with *Donaciaclavipes*, which occurs on the same food plant, the numbers of *P.bracata* specimens are also much fewer. Recent records are extremely rare.

###### Distribution.

All parts of Europe except southern Europe and north Scandinavia, continuing to central Asia, including southern parts of Russia and western Siberia. Records exist for Europe: Austria, Belgium, Bosnia-Herzegovina [new in PalCat], Belarus, Bulgaria, Croatia [first record], Czech Republic, Denmark, Estonia, Finland, France, Germany, Great Britain, Hungary, Ireland, Italy, Latvia, Lithuania, Luxembourg, Moldavia, Montenegro [first record], The Netherlands, Norway, Poland, Romania, Russia (central and south parts of European Russia), Serbia [new in PalCat], Slovakia, Slovenia, Sweden, Switzerland, Ukraine.

Asia: Azerbaijan, China (Xinjiang [first record]), Georgia, Iran [new in PalCat], Kazakhstan, Kyrgyzstan [new in PalCat], Russia (south Siberia [new in PalCat], west Siberia).

###### New country records additional to Silfverberg (2010).

Bosnia-Herzegovina: Mohr (1966b).

Croatia • 3 ex.; Dalmatia; E. Geiser 2019 det.; coll. Frey in NHMB.

Montenegro • 2 ex.; Montenegro; E. Geiser 2019 det.; coll. Frey in NHMB.

Serbia: Gavrilović and Ćurčić (2011) and Mohr (1966b).

China • 2 ex.; Xinjiang, “Ost-Turkestan, Bagratsch-Kul” [Bosten-Lake], Kurla; May 1902; Hauser leg.; E. Geiser 2019 det.; HNHM • 1 ex.; Xinjiang, Kuldscha province, Upper Ili valley [“Ober Jli-Thal”]; 1897; F. Hauser leg.; “*Pl.braccata*” H. Goecke 1956 det., E. Geiser 2019 vid.; coll. Frey in NHMB. Note: Bosten-Lake lies east of Kurla; Kuldscha is now called Yining (in Chinese). Both Bosten-Lake and Kuldscha are located in north-western Xinjiang, on the northern side of the Ili River in the Dzungarian basin, ~ 70 km east of the border with Kazakhstan.

Iran • 2 ex.; Khorasan-e Razavi province, Sabzevar; 36°12′N, 57°35′E; 1024 m a.s.l.; 23. Aug 2012 (Samin 2018).

Kyrgyzstan: Bieńkowski (2014).

Russia: South Siberia (Gus’kova et al. 2018).

###### Material examined.

More than 100 specimens from different localities throughout the distribution area.

##### Plateumaris consimilis

Taxon classificationAnimaliaColeopteraChrysomelidae

(Schrank, 1781)

97C4226F-A4DA-5EF7-9908-E3122FEBD083

Fig. 5

Leptura consimilis Schrank, 1781: 155.
Plateumaris consimilis f. aerea Bechyné, 1942: 234, 236 [infrasubspecific name].
Leptura assimilis Schrank, 1781: 156.
Plateumaris consimilis f. coerulea Bechyné, 1942: 234, 236 [infrasubspecific name].
Donacia discolor Hoppe, 1795: 45 [homonym].
Plateumaris consimilis f. flavipes Bechyné, 1942: 234, 236 [infrasubspecific name].
Plateumaris consimilis f. nigripes Bechyné, 1942: 234, 236 [infrasubspecific name].
Donacia rufipes Olivier, 1791: 292.
Plateumaris tenuicornis Balthasar, 1934: 128 [syn. nov.].
Donacia variabilis Kunze, 1818: 39.
Plateumaris consimilis f. violacea Bechyné, 1942: 234, 236 [infrasubspecific name].
Plateumaris consimilis f. viridis Bechyné, 1942: 234, 236 [infrasubspecific name].

###### Type locality.

*Plateumarisconsimilis*: unknown, but possibly in Austria (the country in 1781 was much larger than today) because the original description is in a book titled ‘Enumeratio insectorum Austriae indigenorum’.

###### Type material.

Holotype or type series of *P.consimilis* unknown.

**Figure 5.**
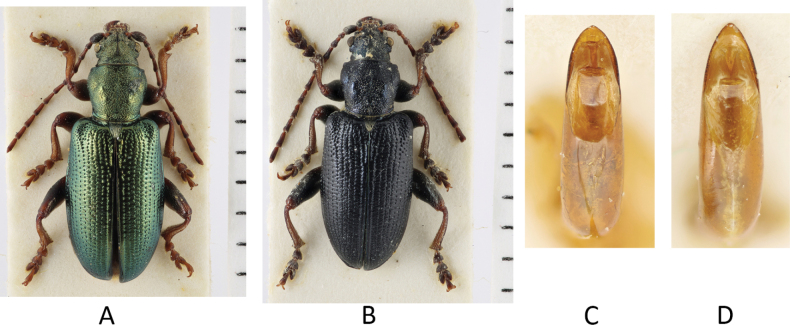
*Plateumarisconsimilis*: Variability of colours and of the shape of the median lobe **A, B** habitus **C, D** median lobe (photographs by K. Matsumoto). Scale bars: one unit – 1 mm.

###### Taxonomic history and synonymies.

Bechyne (1942) detailed statistics about the different colours and subtle structures on the pronotum of *P.consimilis*, but without convincing results. He named colour variations as “forma” but conceded that there also existed combinations of colours in between. These names are indicated above for the sake of completeness, but they are irrelevant to systematics.

Schrank de Paula (1781: 155) described *Lepturaconsimilis* with bronze and black-golden colouration. On the next page of this publication, he described a new species *Lepturaassimilis*. The difference from the former species is indicated as “black” and the elytra with nine rows of punctures in contrast to those of *P.consimilis* which he described with ten rows of punctures [both species have elytra with 11 rows of punctures]. In that pioneer period, this species common in central Europe was also described as *Donaciarufipes* by Olivier (1791), *Donaciadiscolor* by Hoppe (1795), and *Donaciavariabilis* by Kunze (1818), who already mentioned the great variability of this species by its specific name.

*Donaciadiscolor* was described by Hoppe (1795). According to Bousquet (2016) this was published on [30] April 1795, but a description of a *Donaciadiscolor* had been already published by Panzer on 14 February 1795 (Bousquet 2016). Therefore, *Donaciadiscolor* Hoppe was immediately a homonym. Later, both species were assigned to *Plateumaris*, therefore *D.discolor* Hoppe remained a homonym.

*Plateumarisconsimilisorientalis* was described by Shavrov (1948) (see Geiser and Geiser 2023), which he thought to represent a subspecies of *P.consimilis*, but it resulted in being synonymous with *P.weisei* (see at *P.weisei* below for further explanation).

###### Diagnosis.

Pronotum cordate, anterior tubercles slightly convex. Upper side with metallic lustre in varying colours, antennae, and legs at least partly reddish brown. Elytra 1.5–1.8× longer than wide.

###### Description.

***Size***: 6.0–9.2 mm.

***Colour***: Very variable, upper side greenish, bluish, cupreous, bronze, or black with metallic lustre, some black specimens almost without metallic lustre (Fig. 5A, B).

***Head***: Same colour as pronotum. Antennae approximately half the body length or slightly longer, Antennomeres reddish brown at least at the basal part, the apical part can be darkened. ½ A1 ≥ A2, ½ A3 ≥ A2, A4 ≥ A3, A1 ≈ A4, A5 … A11 ≈ A4.

***Pronotum***: cordate, ahead wider than behind, anterior tubercles only slightly convex, disc uniformly punctate, median line obsolete to fine but distinct.

***Elytra***: 1.5–1.8× longer than wide, never twice as long as wide, side contours slightly convex, not parallel, elytra rugose and punctulate.

***Legs***: Colour variation from completely reddish brown to only reddish at the joints, femora basally very broad, metafemora with sharp or broad tooth.

***Aedeagus***: The shape varies between the short, more rounded form in *P.bracata* and the elongated acute form of *P.rustica* (Fig. 5C, D).

The most similar species are *Plateumarisbracata* and *P.rustica*: *P.bracata* has longer elytra, ~ 2× as long as wide and the side contour of the elytra is parallel, not convex (Fig. 4C). *Plateumarisrustica* has the pronotum not cordate, and disk and tubercles flattened (Fig. 10A). *Plateumarisweisei* could be mistaken for *P.consimilis* but their distribution areas hardly overlap.

###### Biology.

Although *P.consimilis* is one of the common *Plateumaris* species, its larva was not described until 2014 by Medvedev and Muravitsky. The larvae and adults feed on *Carex* sp. (Cyperaceae). Also, *Juncusarticulatus* (Juncaceae) and *Caltha* sp. (Ranunculaceae) are mentioned as food plants of adults, on which they feed on the pollen (Rheinheimer and Hassler 2018). *Plateumarisconsimilis* lives in wetlands, fens, near springs, and moor grass meadows. It is more frequent on low calcareous soils, where it is usually the only species of Donaciinae. It is site-loyal and cannot be caught in pitfall traps (with rare exceptions) (Krause 1982; pers. obs.).

###### Distribution.

Western Palaearctic: mainly continental Europe up to southern Sweden, very rare in south and east Europe and west Siberia. Records exist for: Europe: Albania [first record], Austria, Belarus, Belgium, Bosnia-Herzegovina, Bulgaria, Croatia, Czech Republic, Denmark, France, Germany, Hungary, Italy, Latvia, Liechtenstein [new in PalCat], Lithuania, Luxembourg, The Netherlands, North Macedonia [new in PalCat], Poland, Romania, Russia (central part of European Russia), Serbia [new in PalCat], Slovakia, Slovenia, Spain, Sweden, Switzerland, Ukraine.

Asia: Georgia [first record], Russia (west Siberia), Turkey [new in PalCat].

###### New country records additional to Silfverberg (2010).

Albania [first records] • 3 ex.; Qarku i Kukësit, Kula e Lumës, “Albanien Expedition, Kula Ljums”; 18–28 May 1918; H. Goecke 1956 det., E. Geiser 2019 vid.; NHMB [ex coll. Frey] • 1 ex.; Qarku i Kukësit, Gjallica e Lumës, “Albanien Expedition, Gjalica Ljums”; 17–26 Jun 1918; H. Goecke 1956 det., E. Geiser 2019 vid.; NHMB [ex coll. Frey].

Liechtenstein: Walter (1990).

North Macedonia: Gruev and Tomov (1984).

Serbia: Gavrilovic and Curcic (2011).

Georgia [first record] • 2 ex.; Mtskheta, “Transcaucasia, Mazcheta, pr. Tiblisi”; 4–23 Jun 1987; Wrase and Schülke leg.; E. Geiser 2019 det.; NHMB.

Turkey: Ekiz et al. (2020).

###### Remarks.

*Plateumarisconsimilis* does not occur in the East Palaearctic which has also been confirmed recently by Hayashi (2020). Records from Far East and Japan, e.g., in Goecke (1960) or Warchałowski (2010), are due to records of “*Plateumarisconsimilisorientalis* Shavrov, 1948” which is synonymous with *P.weisei* (see below). Note that specimens of *P.weisei* misidentified as *P.consimilis* were found in collections (pers. obs.).

###### Material examined.

More than 200 specimens from different localities throughout the distribution area.

##### Plateumaris tenuicornis

Taxon classificationAnimaliaColeopteraChrysomelidae

Balthasar, 1934 syn. nov.

679487A5-B255-548F-98FE-A0947B3A56B7

Fig. 6

###### Type locality.

Bosnia-Hercegovina: Dol. Tuzla, Bosnia.

###### Type material.

**Figure 6.**
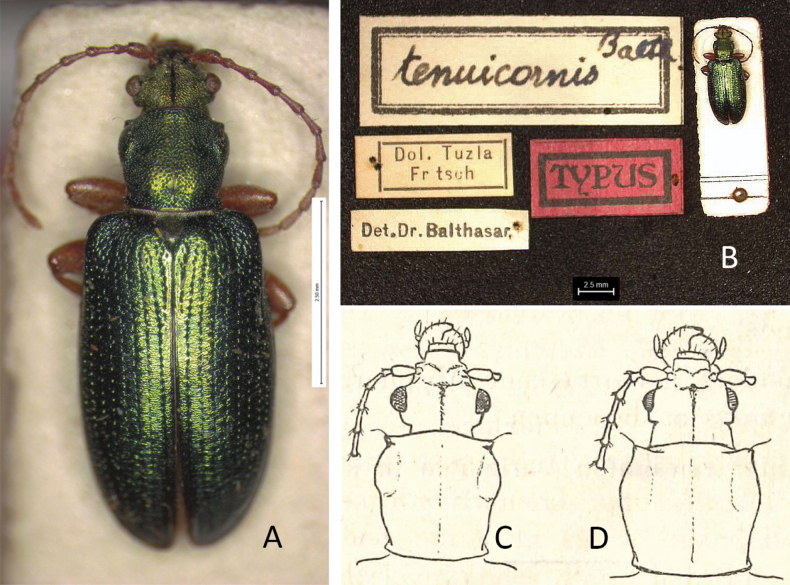
*Plateumaristenuicornis* Holotype **A** habitus **B** labels of Holotype (photographs by M. Sečanský), comparison of the pronotum of **C***P.tenuicornis* with **D***P.consimilis* (from the original description of *P.tenuicornis* by Balthasar 1934: 128). Scale bars: 2.5 mm.

***Holotype*** of *Plateumaristenuicornis*. Bosnia-Herzegovina • 1 ex.; Bosnia, Dol. [Dolina?] Tuzla; Em. Fritsch leg.; SNMC. Fig. 6A, B. I examined the holotype in 2020 and it is the only specimen known.

###### Remarks.

Balthasar (1934) described a species *Plateumaristenuicornis* from one *P.consimilis* specimen collected in Bosnia, which he studied in the collection of the Slovak National Museum, Bratislava. The sketches where he compared the pronotum of both species are provided in Fig. 6C, D. Bechyné (1942) published an article about this description. He studied 335 specimens of *P.consimilis*, mostly from the area which belongs now to the Czech Republic, but also from central France and Podolia, a historic region in Eastern Europe located in the west-central and south-western parts of Ukraine and north-eastern Moldova, but he did not study the holotype of *P.tenuicornis* (possibly due to the political situation in Europe at that time). He meticulously worked out that all described characters of *P.tenuicornis* were within the variation range of the characters of *P.consimilis*. Bechyné published this article in Czech and Latin in a Czech journal, which has been ignored by most Donaciinae specialists. The English translation of the Latin text is now available in Geiser and Geiser (2023).

Askevold (1991) knew neither of the article of Bechyné (1942) nor of the holotype of *P.tenuicornis*, but he studied the description of Balthasar (1934) and concluded “All character states used by Balthasar are ones that I have also found among *P.consimilis*” and then declared *P.tenuicornis* as a probable new synonym. I studied the holotype of *P.tenuicornis* still stored in the Slovak National Museum in Bratislava (Fig. 6A, B) and I can confirm that Bechyné and Askevold were correct in every detail.

Because Bechyné only indirectly treated *P.tenuicornis* as a synonym, and because Askevold only suggested that *P.tenuicornis* should be considered as a probable new synonym, I determined that *P.tenuicornis* Balthasar, 1934 is a new synonym of *P.consimilis* (Schrank, 1781) according to Bechyné (1942), supposed by Askevold (1991), and now confirmed based on a study of the type material and original descriptions.

##### Plateumaris constricticollis

Taxon classificationAnimaliaColeopteraChrysomelidae

(Jacoby, 1885)

3BB0CA03-1259-5ACD-A909-E7F14847D05B

Fig. 7

Donacia constricticollis Jacoby, 1885: 192.
Plateumaris constricticollis babai Chûjô, 1959: 2.
Donacia constricticollis constricticollis Jacoby, 1885: 192 (= Plateumarisconstricticolliskurilensis L. N. Medvedev, 1978b: 83).
Plateumaris constricticollis toyamensis Tominaga & Katsura, 1984: 27 (= Plateumarisconstricticollischugokuensis Tominaga & Katsura, 1984: 28).

###### Type localities.

*Plateumarisconstricticollis*: Japan; *P.constricticollisbabai*: Honshu: Niigata Prefecture, Yoshigahira, Shitada-mura; *P.constricticollistoyamensis*: Tsubura-ike, alt. 690 m, Kamiichi-machi, Naka-niikawa-gun, Toyama prefecture; *P.constricticollischugokuensis*: Koiga-kubo, alt. 570 m, Tessei-cho, Atetsu-gun, Okayama prefecture.

###### Type material.

***Holotype*** of *P.constricticollis*: Japan • 1 ♂; Hokkaido, Lake Junsai, N of Hakodate; 43°7'N, 145°6'E; 28–30 Jul 1880; G. Lewis leg.; BMNH 1910-320. The holotype was examined in 2019.

***Holotype*** of *P.constricticollisbabai*: Japan • 1 ♀; Honshu, Niigata Prefecture, Yoshigahira in Mt. Sumon; 25 Jun 1955; K. Baba [leg.]; “*P.constricticollisbabai* Chȗjȏ” M. Chȗjȏ 1959det.; KUEC.

***Holotype*** of *P.constricticollistoyamensis*: Japan • 1 ♂; Honshu, Toyama-Prefecture, Kamiichi-machi, Naka-niikawa-gun, Nakanomata, Tsubura-ike; 690 m a.s.l.; 20 Jun 1983; K. Katsura leg; OMNH TI-17.

***Holotype*** of *P.constricticollischugokuensis*: Japan • 1 ♂; Honshu, Okayama prefecture, Tessei-cho, Atetsu-gun, Koiga-kubo; 570 m a.s.l.; 13 Jun 1982; O. Tominaga leg.; OMNH TI-18.

###### Taxonomic history and synonymies.

Jacoby (1885) described this *Plateumaris* species as *Donaciaconstricticollis*. The details of the location and the date are not tagged to the holotype, and the label contains only “Japan G. Lewis, BMNH 1910-320”. Bates (1883) published the itinerary of G. Lewis’ journey through Japan from February 1880 to September 1881 that contains the exact data. The type specimen was collected at Lake Junsai near Hakodate in south Hokkaido where Lewis sojourned 28–30 July 1880.

Lays (2002) refers to a female type specimen: 1 ♀, “Type”, “*Donaciaconstricticollis* Jac.” [no further label data, origin unknown], stored in RBINS. It is unlikely that this “type” specimen could be a paratype or allotype of the series collected in Hokkaido at the same site as the holotype. According to Lays (2002) this specimen belongs to the subspecies *P.constricticollisbabai*, which occurs only in central Honshu. One must keep in mind that some decades ago “type” labels were sometimes added later to specimens in several museum collections without thorough studies.

Specimens of *P.constricticollis* reveal a remarkable variation in body size and colouration, pronotal disc, and even genital structures. This resulted in the description of four subspecies. Further studies concluded that there were two subspecies in addition to the nominate species, and therefore the other two subspecies names are synonyms (Hayashi and Shiyake 2004). This is also confirmed by several molecular studies (Sota and Hayashi 2007; Sota et al. 2007; Sota et al. 2008; Hayashi and Sota 2014). However, the morphological discrimination of these subspecies is very difficult because of the variations in some key characters.

Chûjô (1959) was the first to describe a subspecies, *P.c.babai* from Niigata Prefecture. In 1978, Medvedev described *P.c.kurilensis* from Kunashiri, the southernmost Kurile Island, near Hokkaido. This subspecies was synonymised with *P.c.constricticollis* by Hayashi and Shiyake (2004: 114). Tominaga and Katsura (1984) described the two subspecies *P.c.toyamensis* and *P.c.chugokuensis*, and the latter was synonymised with the former by Hayashi and Shiyake (2004: 116).

###### Diagnosis.

**Figure 7.**
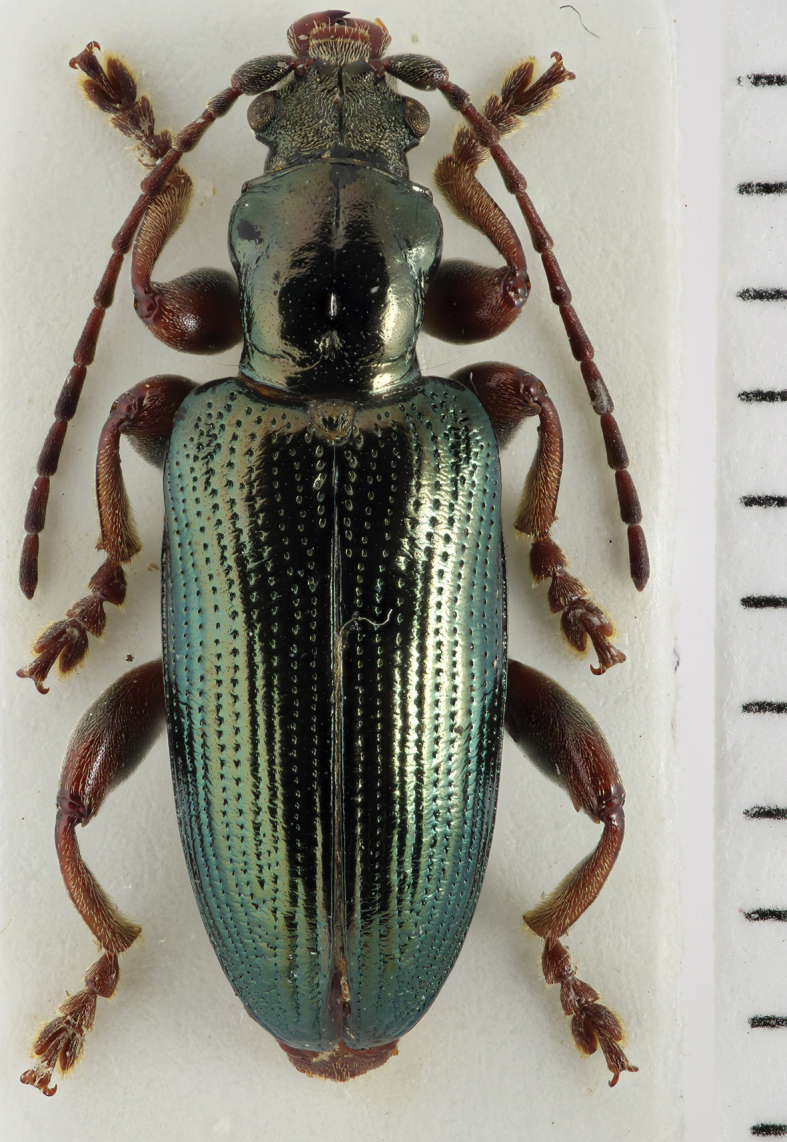
*Plateumarisconstricticollis* Habitus (photograph by K. Matsumoto). Scale bar: one unit – 1 mm.

The characters common to all subspecies are the following: surface very shiny (Fig. 7), dorsal colouration variable, bronze brown or dark cupreous, black, blue, or green, pronotum cordate, slightly longer than broad, anterior tubercles prominent, elytra regularly and strongly punctate, metafemora with blade-like tooth. It looks similar to a glossy *P.consimilis* that does not occur in the East Palaearctic region.

###### Description.

***Size***: 6.6–11.9 mm.

*Plateumarisconstricticollis* and its subspecies have been thoroughly studied by Japanese colleagues, with detailed descriptions of their morphological characters and their variations, as well as phylogeny, biogeographical history, biology, and molecular analyses (Tominaga and Katsura 1984; Hayashi 2002, 2020; Hayashi and Shiyake 2004; Hayashi and Sota 2014). The three subspecies and their distinguishing characters are described in the section “Identification Key”.

The similar species *Plateumarisweisei* is not shiny or glossy, its pronotum not cordate, and the legs are longer and slenderer.

###### Biology.

Sota et al. (2007) analysed the geographic variation in body size and ovipositor dimensions in three subspecies in different climatic conditions and on different host plants, and reported a significant correlation of the body size and ovipositor size with snow depth. The larvae feed on *Carex* sp. (Cyperaceae) (Narita 2003). Cocoons of *P.c.babai* were also found on roots of *Phragmitesaustralis* (Poaceae), *Carexthunbergia*, and *Carexampliata*. *Eleocharis* sp. is recorded as a larval host plant for *P.c.toyamensis* (Sota et al. 2007).

###### Distribution.

*Plateumarisconstricticollis* is endemic to the Japanese Archipelago (Hokkaido, Honshu, and Kunashiri).

*Plateumarisconstricticollisconstricticollis*: southernmost Kurile Island Kunashiri, Hokkaido and northern part of Honshu until prefecture of Yamagata (Medvedev 1978; Tominaga and Katsura 1984).

*Plateumarisconstricticollisbabai*: Honshu: prefectures Fukushima, Niigata, Nagano, Gunma, Tochigi, Ibaraki, and Chiba.

*Plateumarisconstricticollistoyamensis*: Honshu: prefectures Toyama, Gifu, Ishikawa, Aichi, Hyogo, and Okayama.

###### Material examined.

Approximately 60 specimens from Japan.

##### Plateumaris roscida

Taxon classificationAnimaliaColeopteraChrysomelidae

Weise, 1912

1F233C4B-03D3-5022-87C6-4E13DB8DD804

Figs 8 , 9

Plateumaris roscida Weise, 1912: 77.
Plateumaris annularis Reitter, 1920: 41.

###### Type localities.

*Plateumarisroscida*: Russia, Transbaikalia (Zabaykalsky Krai): Tschita; *Plateumarisannularis*: Russia, Far East, Khabarovsk Krai, Nikolayevsk-on-Amur.

###### Type material.

***Holotype*** of *P.roscida*: Russia • 1 ♂; East Siberia, Transbaikalia, Tschita; ZMHB. Label text: “Typus [red]// roscida m. //coll. J. Weise //Zool. Mus. Berlin //Holotype ♂ *Plateumarisroscida* Weise [red]//*Plateumarisroscida* Weise det. I.S. Askevold 1989”.

No location label is tagged to the holotype, but the type locality “Tschita” is indicated in the original description by Weise (1912). The holotype was examined in May 2023.

***Holotype*** of *Plateumarisannularis*: Russia • 1 ex.; Far East, Khabarovsk Krai, Nikolayevsk-on-Amur, “Amur region, Chabarowsk, Nikolajewsk”; L. Graeser leg.; depository unknown. At first stored in coll. W. Koltze, current depository presumably SDEI, but needs confirmation.

###### Taxonomic history and synonymies.

Weise (1912) described *Plateumarisroscida* (see Geiser and Geiser 2023) based on a specimen from Tschita. He obtained it from Johann Nepomuk Ertl (1860–1925, Munich, Germany), a dedicated beetle collector who had connections to missionaries. This specimen was presumably collected during a journey to China.

Reitter (1920) published an identification key for Palaearctic Donaciinae. In a footnote to *P.sericea* he described *P.annularis* from Russia, Far East, Amur region, Chabarowsk (krai), Nikolajewsk, coll. Koltze (see Geiser and Geiser 2023). Reitter did not mention in the description that this *P.annularis* is armed with a prominent metafemoral tooth, but he provided this information indirectly two paragraphs later when he contemplated if *P.obsoleta* could be the same species as *P.annularis*. He also described that, in contrast, *P.obsoleta* has completely dark legs and the posterior femora are practically unarmed, or only bluntly angled. This description and further notes in Reitter (1920) match completely with the characters of *P.roscida*.

Kolossow (1930: 29) synonymised *P.annularis* with *P.roscida* with the laconic line “*Plateumarisannularis* Reitt. (1920) = *Pl.roscida*Weise (1912)”. Goecke (1957a) studied a specimen from ZSM, labelled “Samml. Ertl” [= collection of Ertl] and “*Pl.roscida* n. sp. Wse” in the handwriting of Weise. He also examined four specimens from the then “Deutsches Entomologisches Institut Berlin” (now SDEI) labelled “*Pl.annularis* Reitter”, one of them labelled “Nikolajewsk, Graeser” and “*Pl.annularis* Rtr., Chabaroska, Weise” and another two specimens labelled “*Pl.annularis*” without location labels. All four specimens were labelled as “Type”. He then compared the *P.roscida* specimen with the *P.annularis* specimens and confirmed the statement of Kolossow (1930) that they all belong to *P.roscida*.

Askevold (1991) studied other specimens from Russia and north China identified as *P.annularis* and confirmed that they belonged unambiguously to *P.roscida*. He also suggested that *P.caucasica* may be synonym of *P.roscida* because of the description of Zaitzev (1930), but he conceded that the geographic distance between the Caucasus and Transbaikalia caused a problem.

**Figure 8.**
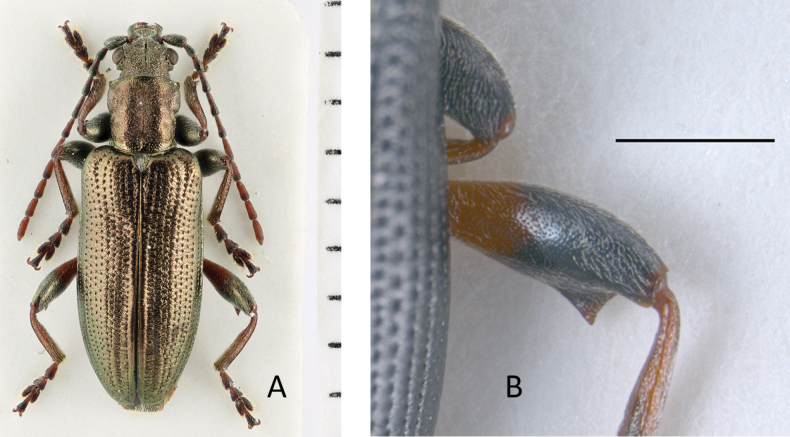
*Plateumarisroscida***A** habitus (photograph by K. Matsumoto) **B** metafemur with typical double colouring and prominent tooth (photograph by E. Geiser). Scale bars: one unit – 1 mm (**A, B**).

**Figure 9.**
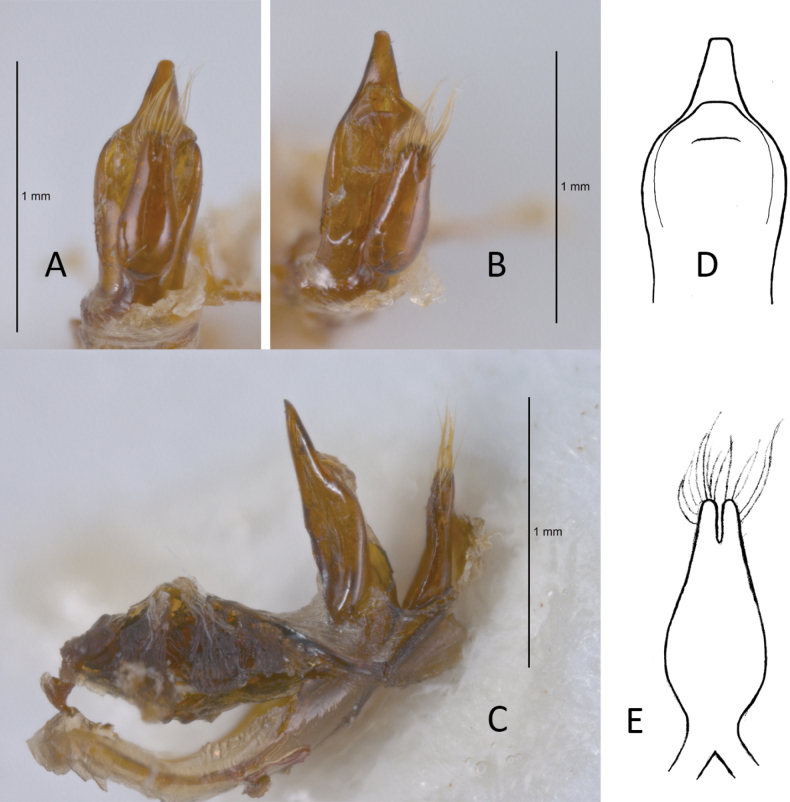
*Plateumarisroscida* aedeagus, photographed from different angles and drawings in frontal view **A, B** specimen from Primorsky Krai (ZMS) **C** specimen from Amur Oblast (NHMB) **D** median lobe **E** cap of tegmen (**A–C** photographs by E. Geiser, **D, E** drawings by G. Geiser). Scale bars: 1 mm.

###### Diagnosis.

Upper side bronze, bluish, or purplish, flat-lustrous, habitus similar to *P.sericea*, antennomeres reddish basally and darkened apically, pronotum with flattened anterior tubercles, femora reddish on basal half and dark on apical half. Aedeagus with a conspicuous elongated peak.

###### Description.

***Size***: 6.7–9.7 mm.

***Colour***: Bronze or dark with bluish or purplish lustre.

***Antennae***: Slender, annulated, antennomeres basally rufous, apical dark or metallic, A2 < A3 < A4.

***Pronotum***: Almost quadratic or slightly longer than wide, anterior tubercles flattened, disc coarsely and closely punctate with fine wrinkles, median groove narrow or indistinct, in posterior part short and slightly deeper, then forked into two horizontal grooves near the bottom line.

***Elytra***: Oblong, with shallow impressions, coarsely and densely rugose on most of surface, punctures regular, strong, and deep, interstices wrinkled, interstices ~ 2–4× puncture diameter.

***Legs***: In most specimens the femora are reddish on basal half and dark metallic on apical half, tibia reddish with dark parts, tarsomeres dark or with reddish basal part. Some specimens with entirely reddish legs. Metafemora with prominent, thorn-shaped tooth in most specimens.

***Pygidium*** of females with an apical notch, males broadly emarginate.

***Aedeagus***: Median lobe with a conspicuous elongated peak, cap of tegmen with a deep apical notch (Fig. 9).

Two similar species are *Plateumarissericea* and *P.shirahatai* which differ: in *P.roscida* the pronotum and its tubercles are flattened, legs with large reddish parts, and the aedeagus has a conspicuous elongated peak.

###### Biology.

Bieńkowski 2014 mentions: *Carex* sp. (Cyperaceae) as host plants. The larva has not yet been described.

###### Distribution.

East Palaearctic: East of Lake Baikal to Far East, the Sakha (Yakutia) Republic, Amur region in Russia and north-east China (Harbin, Heilongjiang). Records exist for Asia: northern China (Heilongjiang, Inner Mongolia) [new in PalCat], Russia (East Siberia, Far East).

###### New country records additional to Silfverberg (2010).

China – Heilongjiang • 1 ex.; Harbin; 3 Jul 1952; E. Geiser 2021 det.; BM1953-715, (BMNH). • 2 ex.; Xinkai (Khanka) Lake, Bathing beach area; 45°21'52"N, 132°18'55"E; 11 Jun 2018; among strandline detritus; R.B. Angus, F.L. Jia, Z.L. Liang leg., E. Geiser 2021 det.; BMNH.

China: Heilongjiang and Inner Mongolia: Askevold (1991).

###### Material examined.

More than 30 specimens from different localities throughout the distribution area.

##### Plateumaris rustica

Taxon classificationAnimaliaColeopteraChrysomelidae

(Kunze, 1818)

12361C83-9147-5417-95B5-09BFBCBB26E6

Fig. 10

Donacia rustica Kunze, 1818: 31.
Donacia abdominalis Bedel, 1891: 218 [nomen nudum].
Donacia affinis Kunze, 1818: 37.
Plateumaris rustica ab. forojulensis Gortani, 1906: 20 [infrasubspecific name].
Donacia fusca Zschach, 1788: 27 [nomen oblitum].
Donacia pallipes Kunze, 1818: 35.
Plateumaris rustica var. picipes Weise, 1898: 180.
Donacia planicollis Kunze, 1818: 34.
Donacia rustica Schüppel, 1818: 31 [nomen nudum].
Plateumaris sulcifrons Weise, 1900: 267 [syn. nov.].

###### Type locality.

Germany, surroundings of Berlin [Kunze, 1818: 31: “in der Gegend von Berlin”].

###### Type material.

Type specimens missing.

###### Taxonomic history and synonymies.

Kunze (1818) described four new *Donacia* species (see original text and translation in Geiser and Geiser 2023) which in fact belong to one single *Plateumaris* species (Askevold 1991 in part). The name *Donaciarustica* was described first in this publication, so *planicollis*, *pallipes*, and *affinis* are now synonyms.

Some authors, like Jolivet (1970) and Borowiec (1984), cited the authority of *P.rustica* as Schüppel (1818, in Kunze 1818) but this is inaccurate since Kunze (1818) wrote after the description: “*D.rustica* Schüppel in litt.” Therefore, the suggestion is there that the name is derived from J. Schüppel (Berlin), but Kunze actually described this species and published it. The unambiguous authority of *P.rustica* is Kunze; therefore, *D.rustica* (Schüppel, 1818) is a nomen nudum. Note that there is no publication of “Schüppel (1818)”.

The names *P.abdominalis* Bedel, 1891 and P. (Donacia) abdominalis Olivier, 1795 [1800 is correct, see above for *P.bracata*] were erroneously attributed to *P.rustica*. The name *P.abdominalis* is frequently mentioned as a synonym for *P.rustica* or *P.affinis* as occurs in the key by Jacobson (1892): “*abdominalis* Oliv.” with *P.affinis* as its synonym. Clavareau (1913) defined “*abdominalis* Bedel” as synonymous with *P.affinis* and this was followed by Reitter (1920), Winkler (1930), Goecke (1960), and Jolivet (1970); the latter also mentioned “*abdominalis* Olivier” as synonymous with *P.bracata*, but Olivier did not describe it [see above for *P.bracata*]. Also, Bedel (1891) did not describe *P.abdominalis*; in his list of the Coleoptera of the Seine basin he mentioned *P.abdominalis* Olivier, together with the synonyms *affinis* Kunze, 1818 and [sic!] *fusca* Zschach, 1788 (synonymous with *P.bracata*). Therefore, *P.abdominalis* Bedel is a nomen nudum, a misidentification or misinterpretation by Bedel, but not a synonym of *P.affinis*.

*Donaciaaffinis* was also described in Kunze (1818) (see Geiser and Geiser 2023). Goecke (1943) suggested that *P.affinis* should be considered synonymous with *P.rustica*. Askevold (1991: 37) synonymised it after examination of ~ 250 specimens from various locations in Europe. These beetles are typically separated in keys by the colour of the antennae, legs, and ventral side, and by the metafemoral tooth size, but these are highly variable characters among many Donaciinae (pers. obs.). In fact, the aedeagi of these two “species” are indistinguishable.

*Plateumarisforojulensis* was described by Gortani (1906) as aberration.

*Donaciafusca* was regarded as synonymous with *P.affinis*, but it is a nomen oblitum (Jolivet 1970).

*Plateumarispallipes* was assigned as a synonym of *P.affinis* and *P.planicollis* as a synonym of *P.rustica*. As the original descriptions of Kunze (1818) show, all characters are within the variation range of the typical characters of *P.rustica* (Geiser and Geiser 2023).

*Plateumarispicipes* was described by Weise (1898) as a variation (Geiser and Geiser 2023). It refers to specimens with at least very dark femora up to very dark legs. Albeit the basal joints of the femora are always reddish.

###### Diagnosis.

**Figure 10.**
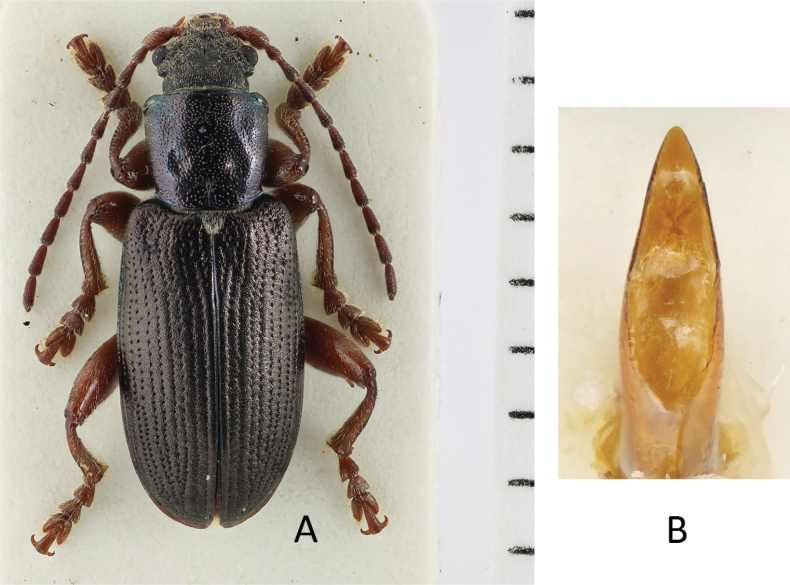
*Plateumarisrustica***A** habitus **B** median lobe (photographs by K. Matsumoto). Scale bar: one unit – 1 mm.

Upper side mostly metallic, antennae and legs entirely or partly reddish brown. It has a very smooth and the most flattened pronotum of all Palaearctic *Plateumaris* species (Fig. 10A).

###### Description.

***Size***: 6.5–9.0 mm.

***Colour***: Upper side bronze or black with greenish, bluish, or purplish metallic lustre, colour of pronotum and elytra mostly the same but can also differ significantly. Antennae and legs entirely or partly reddish brown.

***Head***: Frons with deep or shallow groove, longitudinal calli distinctive or flattened.

***Antennae***: Filiform, each antennomere yellow or reddish at the basis, darkened at the apex, extent of darkened zone very variable, 2^nd^ antennomere 2–3× smaller than other antennomeres which are approximately equal in length, only the 3^rd^ antennomere is sometimes slightly smaller than the others: (2–3)× A2 = A1 = A4 … A11; A3 ≤ A4.

***Pronotum***: Almost quadratic, only at the basis slightly constricted, with flat disc and indistinct anterior tubercles; surface shiny or alutaceous, disc smooth with small shallow dots, more or less densely dotted, median line varies from imperceptible to distinctive.

***Elytra***: Punctures very delicate, interstices with slight transversal rugae, interstices 2–4× puncture diameter. Ratio of elytral length to width: 1.7–2.0.

***Legs***: Yellow reddish, sometimes partly or almost entirely darkened, piceous, but always with reddish joints (var. picipes Weise, 1898). Femora basally broad, metafemoral tooth very variable, mostly prominent, in some (mostly female) specimens very small or imperceptible. There is no geographic correlation concerning the size of the tooth.

***Aedeagus***: Median lobe distinctly elongated, apex acute (Fig. 10B).

There are two similar species. *Plateumarisconsimilis* has the pronotum distinctly cordate and the disc is not flattened. The pronotum of *P.weisei* is trapeziform and slightly longer than wide. In the territories where their distribution areas are overlapping (European part of Russia) it can be distinguished from *P.weisei* by the quadratic shape of the pronotum.

Also, the aedeagi of these species are clearly different (compare Fig. 10B with Fig. 17B, C, E).

###### Biology.

The larvae are oligophagous on *Carex* sp. and other Cyperaceae. Adults feed on leaves and stems, not on pollen (Rheinheimer and Hassler 2018). For identification of the larvae see Steinhausen (1994) and Bieńkowski and Orlova-Bieńkowskaja (2004). Although *P.rustica* is widespread in the West Palaearctic region and there are many of its food plants available, it is rather rare suggesting that it needs not only wetland with Cyperaceae but also additional ecological conditions.

###### Distribution.

West Palaearctic region: throughout Europe, further in Algeria, Turkey, Iran, and west Siberia. Records exist for Europe: Austria, Belarus, Belgium, Bosnia-Herzegovina [new in PalCat], Bulgaria, Croatia, Czech Republic, Denmark, Estonia, Finland, France, Germany, Great Britain, Hungary, Ireland, Italy, Crimea [new in PalCat], Latvia, Liechtenstein, Lithuania, Luxembourg, Montenegro [first record], The Netherlands, Norway, Poland, Romania, Russia: northern, central, and southern parts [new in PalCat] of European Russia), Serbia [new in PalCat], Slovakia, Slovenia, Spain, Sweden, Switzerland, Ukraine.

North Africa: Algeria.

Asia: Iran [first record], Russia (west Siberia), Turkey [new in PalCat].

###### New country records additional to Silfverberg (2010).

Bosnia-Herzegovina: Mohr (1966b).

Crimea: Listed in Catalogue: Beetles of the Krym (pers. comm. S. Mosiakin 2019).

Montenegro • 11 ex.; Poljane north-west of Podgorica “Pojane”; *P.rustica* E. Geiser 2019 det.; NHMB [ex coll. Breit in coll. Frey]. Remark: Some specimens were previously identified as *P.forojulensis* (1 ex.) and *P.picipes* (4 ex.).

Russia • 1 ex.; Southern European territory, town Samara Nikolayevsky Uyezd; May 1916; Bostanzhoglo leg.; Zoological Museum of Moscow State University, Russia. Remark: private record by Bieńkowski 2016.

Serbia: Gavrilović and Ćurčić (2011), Mohr (1966b).

Iran • 1 ex.; “Persien, Elbrus Gebirge” [Elbrus mountains]; *Donaciaaffinis* H. Goecke det., *Donaciarustica* E. Geiser 2019 det.; NHMB [ex coll. Reitter in coll. Frey].

Turkey • 25 ex.; Bolu Province, Abant Daği [mountain], Abant Gölü [lake]; 1298 m a.s.l.; 31 May 1999; J. Voříšek and J. Kodada leg.; *P.rustica* E. Geiser 2021 det.; BMNH [ex coll. J. Voříšek]. Bolu province and Kahramanmaraş province (Ekiz et al. 2020).

###### Remarks:

*Plateumarisrustica* was unknown from Turkey. There was no record in the “Checklist of leaf beetles of Turkey” (Ekiz et al. 2013). In 2019 I identified 12 specimens from Bolu province stored in Verona (MSNV) and their detailed data are published in Ekiz et al. (2020), including the type location of *P.sulcifrons* in Kahramanmaraş province. In 2021 I found 25 specimens in the coll. Voříšek which is now stored in BMNH. These specimens were labelled as “*P.sulcifrons* Weise J. Voříšek det.” but are now relabelled as *P.rustica*. Also, the aedeagi of these “*P.sulcifrons*” were identical with the aedeagus of *P.rustica*.

###### Material studied.

More than 200 specimens throughout the West Palaearctic region.

##### Plateumaris sulcifrons

Taxon classificationAnimaliaColeopteraChrysomelidae

Weise, 1900 syn. nov.

0914E4D9-7603-560C-9F85-CBFE096A629A

###### Type locality.

Turkey, Kahramanmaraş province: Süleymanlı “Zeytun” [old name].

###### Type material.

***Holotype***: Turkey • 1 ♀; Kahramanmaraş province, Süleymanlı “Zeytun” [old name]; 37°53'N, 36°50'E; O. Staudinger leg. Probably collected in 1872 (see below for details). Depository unknown.

###### Remarks.

Askevold (1991) declared *P.sulcifrons* as a “probable new synonymy” for *P.rustica*. The holotype (♀) is missing but the analysis of the elaborate original description (see Geiser and Geiser 2023) indicates that the characters of *P.sulcifrons* are within the variation range of *P.rustica* characters (Table 4). Weise indicates a range of [body] length: 8–9 mm and colour variation in the antennae and legs. This suggests that he had examined more than one (female) specimen. Since 1900 the name *P.sulcifrons* is mentioned in almost all catalogues and identification keys for the (West) Palaearctic, but no new records were published.

Besides, there was a confusion about the locus typicus. Weise (1900) published only: “Zeitun (Staudinger)”. This sparse note of the collection site led to misinterpretations: “Zeitun” is correctly assigned to Asia Minor (Reitter 1920; Winkler 1930), but it was misinterpreted by Goecke (1960) as “Żejtun”, a town in the east of Malta. Henceforward it was mentioned as a species from Malta (Jolivet 1970; Borowiec 1984; Askevold 1991; Silfverberg 2010; Warchałowski 2010). This location error confirmed the opinion that *P.sulcifrons* is probably endemic to Malta, therefore missing further records were less suspect.

Otto Staudinger (1830–1900) was a German entomologist who went on numerous collecting trips or promoted them, but insects were not collected in Malta, neither on his own journeys nor on his commissioned trips. In 1872 he visited the Cilician Taurus (Anonymous 1901; Wikipedia [05.10.2022]). Therefore, “Zeitun” mentioned in Weise (1900) is actually “Zeytun” district (now Süleymanlı) of today’s Kahramanmaraş province of Turkey (Ekiz et al. 2020). Different spellings and change of geographical names also did not help to clarify this case.

Unfortunately, the first description does not indicate where these specimens are stored. It is unlikely that Weise returned the type(s) to Staudinger, who was then working on his Lepidoptera Catalogues in his last years. Weise’s private collection and especially the Chrysomelidae part are stored in the Museum für Naturkunde in Berlin (ZMHB), but no *Plateumaris* specimen labelled “*sulcifrons*” could be found there, despite the search by J. Frisch in 2019. I screened the *Plateumaris* collection in 2023 but found no specimen that could be considered the type.

Like other species, where only the type specimen is known, *P.sulcifrons* was suspected to be a synonym of a well-known species. Weise wrote that it is similar to *P.rustica* and *P.affinis*. Askevold (1991) synonymised *P.affinis* with *P.rustica*. He noticed that the characters Weise mentioned are typical for *P.rustica* and suggested that *P.sulcifrons* may be conspecific. Warchałowski (2003) treated *P.sulcifrons* also as synonym to *P.rustica* in his key of the Chrysomelidae of the Europe and the Mediterranean area. However, he treated it as valid species, although doubtful, in his key of the Chrysomelidae of the Palaearctic region (Warchałowski 2010). In Silfverberg (2010) it was listed as valid species from Malta. The locus typicus was corrected to Turkey in Löbl and Smetana (2013).

Unfortunately, I could not examine the type specimen. To confirm the arguments of Askevold (1991) with more details, the characters mentioned by Weise (1900) are compared with the characters of *P.rustica* in Table 4.

The characters which should distinguish *P.sulcifrons* from *P.rustica* are either the same or within the variation range of *P.rustica*. Therefore *P.sulcifrons* is a synonym of *P.rustica*. This was also mentioned in Ekiz et al. (2020) and in Geiser and Bezděk (in press), but without the reason provided here.

**Table 4.** Comparison of the characters of *Plateumarissulcifrons* (as described by Weise 1900) and the corresponding characters of *P.rustica* (by EG).

Characters of *P.sulcifrons*	Characters of *P.rustica* with comments
Length: 8–9 mm	Length of *P.rustica*: 6.5–9.0
Description based on female specimens	Females are usually larger than males in *Plateumaris* species, therefore 8–9 mm matches very well
Slightly convex	Same as *P.rustica*
Upper side bronze-coloured, below jet-black, silky grey	The same colours occur in some specimens of *P.rustica*
Antennae, tibiae and tarsomeres dark reddish	Same as *P.rustica*
Frons with a wide and deep groove	In *P.rustica* frons with deep or shallow groove, narrow or broad, longitudinal calli of every side of the groove distinctive or flattened
Prothorax square, very finely pubescent, very slightly constricted before the base, disc almost flat, shiny, rather densely punctured, middle groove anterior and posterior deepened impressed, the tubercles on both sides almost imperceptible, slightly smooth	These are typical characters of a pronotum of *P.rustica*, the median groove varies from imperceptible to distinctive
Elytra with dotted stripes, interstices narrow	Same as *P.rustica*
Femora unarmed	Tooth of metafemur mostly prominent, in some female [sic!] specimens very small or imperceptible
Similar to *P.rustica* and *P.affinis* but more elongated	Without type specimens it is impossible to estimate what means “more elongated” as the ratio length to width varies in specimens of *P.rustica*

##### Plateumaris sericea

Taxon classificationAnimaliaColeopteraChrysomelidae

(Linnaeus, 1758)

A24795F8-B222-5FD8-B810-E2DD7B3CF048

Figs 11 , 12

Leptura sericea Linnaeus, 1758: 397.
Donacia aenea Olivier, 1791: 292.
Donacia armata Paykull, 1799: 194.
Donacia asiatica Faldermann, 1837: 322.
Donacia sericea var. atropurpurea Westhoff, 1882: 256.
Plateumaris caucasica Zaitzev, 1930: 111 [syn. nov.].
Plateumaris discolor discolor f. coelicolor Bechyné, 1945: 89 [infrasubspecific name].
Donacia comari Suffrian, 1846: 84.
Plateumaris discolor discolor f. cupraria Bechyné, 1945: 89 [infrasubspecific name].
Donacia discolor Panzer, 1795: 216.
Donacia festucae Fabricius, 1792: 116.
Donacia geniculata C. G. Thomson, 1866: 123.
Plateumaris imitatrix Apfelbeck [nomen nudum].
Plateumaris intermedia Apfelbeck, 1912: 239.
Plateumaris discolor kratochvili f. isocoelicolor Bechyné, 1945: 89 [infrasubspecific name].
Plateumaris discolor kratochvili f. isocupraria Bechyné, 1945: 89 [infrasubspecific name].
Plateumaris discolor kratochvili f. isolacordairei Bechyné, 1945: 89 [infrasubspecific name].
Plateumaris discolor kratochvili f. isopurpuricena Bechyné, 1945: 89 [infrasubspecific name].
Plateumaris discolor kratochvili f. isoviolacea Bechyné, 1945: 89 [infrasubspecific name].
Plateumaris discolor kratochvili Bechyné, 1945: 89.
Donacia lacordairii Perris, 1864: 300.
Donacia laevicollis C. G. Thomson, 1866: 125.
Plateumaris sericea ab. levigata Csiki, 1953: 120 [infrasubspecific name].
Donacia sericea var. luctuosa Westhoff, 1882: 256.
Donacia micans Panzer, 1795: 9.
Plateumaris discolor var. nigrita Schilsky, 1908: 603.
Plateumaris nipponensis Nakane, 1963: 18.
Donacia nymphaeae Fabricius, 1792: 116.
? Plateumarisobsoleta Jacobson, 1894: 243.
Donacia palustris Schilling, 1838: 99 [homonym].
Donacia proteus Kunze, 1818: 23.
Plateumaris discolor discolor f. pseudoviolacea Bechyné, 1945: 89 [infrasubspecific name].
Plateumaris discolor discolor f. purpuricena Bechyné, 1945: 89 [infrasubspecific name].
Donacia sibirica Solsky, 1871: 245.
Plateumaris socia S.-H. Chen, 1941: 9.
Plateumaris slovacica Balthasar [nomen nudum].
Plateumaris discolor ab. tatrica Balthasar, 1934: 130 [infrasubspecific name].
Donacia sericea var. tenebricosa Westhoff, 1882: 256.
Donacia violacea Hoppe, 1795: 44 [homonym].
Plateumaris sericea ab. viridis Csiki, 1953: 120 [infrasubspecific name].

###### Type locality and type material.

Because Linnaeus described *sericea* (*Leptura*) in 1758 no type specimen was designated. He stated that it “occurs in Europe” which is correct.

###### Remarks.

*Plateumarissericea* exhibits the highest variability in colour among Donaciinae. The upper side colour is metallic and can be green, golden green, blue, purple, red, violet, bronze, black and all shades in between. This is one of the causes so many “variations” were described which were often used like subspecies names. Additionally, throughout the whole distribution area, some specimens show a reddish base at the antennomeres. Also, few specimens exist with a reddish part near the joints of the femora, tibiae or tarsomeres. In (most) identification keys *P.sericea* is characterised by “antennae and legs entirely metallic“, which is usually correct. Only in recent keys it is mentioned that there can also be reddish parts at some joints. Therefore, these “not entirely metallic” specimens supported the idea that specimens with a reddish spot belong to a different species or at least subspecies. I examined many specimens from the whole distribution area and determined their morphologic characters inclusive of the aedeagus shape are within the variety range of *P.sericea*.

In large European collections, where Asian specimens are stored, many of these Asian specimens show a red base of their antennomeres. Perhaps, these specimens were preferentially collected and stored whereas “entirely metallic“ specimens were considered as common and not worth keeping.

**Figure 11.**
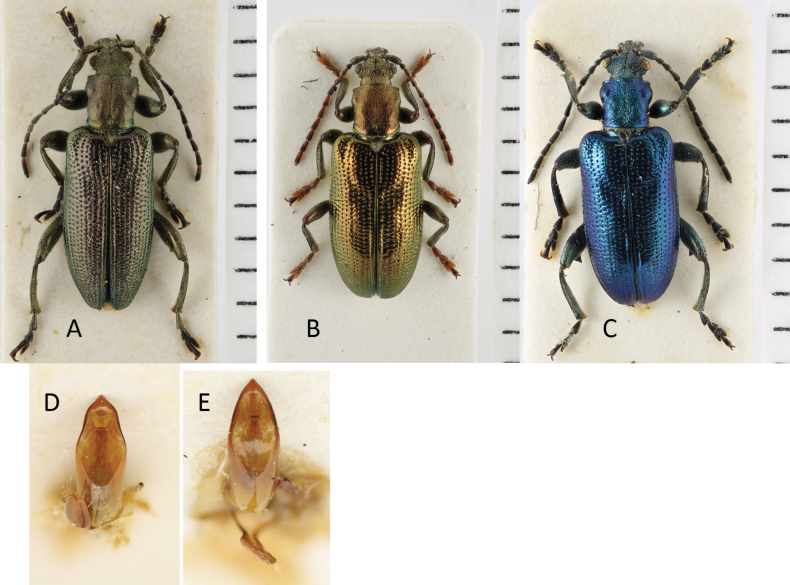
*Plateumarissericea***A–C** habitus illustrating variability in colours, but there are more **D, E** aedeagus: two examples of the variability of the shape of the median lobe (photographs by K. Matsumoto). Scale bars: one unit – 1 mm.

**Figure 12.**
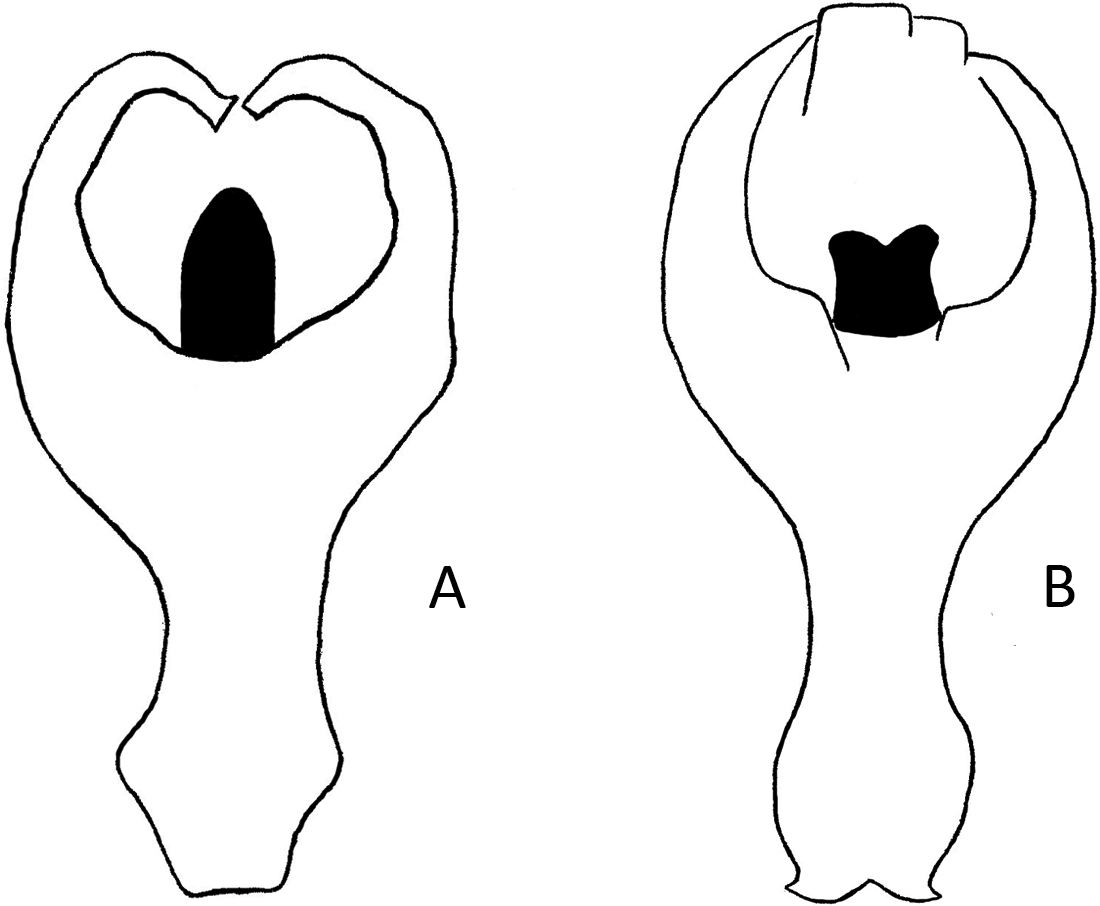
Schematic sketch of the endophallus. Median ejaculatory guide blackened **A***Plateumarissericea* (and specimens identified as *P.discolor*) with apex of median ejaculatory guide rounded. The endophalli of specimens from Poland, Italy and Japan all look the same as in this sketch **B***Plateumarisshirahatai* with apex of median ejaculatory guide notched (drawings by G. Geiser based on photographs from Askevold 1991 and Hayashi 2020).

###### Taxonomic history and synonymies.

The correct data of the first description is *Lepturasericea* (Linnaeus, 1758): 397, and not “Linnaeus, 1760: 196” as it was printed in Silfverberg (2010: 358). See explanation in section “Genus *Plateumaris* C. G. Thomson, 1859, Taxonomic history and synonymies” and in Geiser and Geiser (2023).

The genus name *Donacia* was erected later in 1775 by Fabricius. There he described *Donaciacrassipes* and *Donaciasimplex* and assigned *Lepturaaquatica* L., 1758 to the genus *Donacia*, but, significantly, he did not change the genus name of *Lepturasericea*.

*Plateumarisasiatica* was described as *Donaciaasiatica* by Faldermann (1837) from “Persien” (today’s Iran) and never found again. It was synonymised with *P.sericea* by Kolossow (1929).

*Plateumariscaucasica* Zaitzev, 1930: syn. nov., see below.

Plateumarisdiscolorkratochvilif.coelicolor was described by Bechyné (1945) based on a series of specimens he collected from Přybyslav (central Bohemia). Therefore, *coelicolor* is a published but infrasubspecific name.

Plateumarisdiscolordiscolorf.cupraria was described by Bechyné (1945) in contrast to P.discolorkratochvilif.isocupraria (see below) but both are infrasubspecific names.

*Plateumarisdiscolor* (Panzer, 1795): confirmed synonym, see below.

*Plateumarisimitatrix*: This name with the author “Apfelbeck” can be found on several museum specimens from Bosnia-Hercegovina (HNHM, coll. Frey in NHMB, SDEI), but a description was never published, therefore *P.imitatrix* is a nomen nudum. Viktor Apfelbeck (1859–1934) was a former curator of entomology at the National Museum of Bosnia and Herzegovina, Sarajevo. He labelled several specimens from the Balkans with new names which he regarded as new species. Some of them he described later, some of them not. Nevertheless, some of these specimens were also stored in other museums and can be found nowadays. Goecke (1942a) examined one specimen stored in those days in “Deutsches Entomologisches Institut” in Berlin-Dahlem (now SDEI) and identified it as *P.sericea* unambiguously. I examined three specimens of “*P.imitatrix*” in HNHM and one in NHMB which are also clearly *P.sericea*. Presumably, the reddish base of the antennomeres tempted Apfelbeck to regard it as a new species.

*Plateumarisintermedia* was described by Apfelbeck (1912) on page 239 and not on page 238 in Latin and Serbian (Geiser and Geiser 2023). It was synonymised with *P.sericea* by Goecke (1942b) who examined a specimen labelled “cotype” from Livanskopolje near Livno (Bosnia) stored in those days in “Deutsches Entomologisches Institut” in Berlin-Dahlem (now SDEI).

*Plateumarisdiscolorkratochvili* was described by Bechyné (1945) as a new subspecies in contrast to *P.discolordiscolor*. Both subspecies live in the same habitat. Therefore, they could not be subspecies by definition. The characters to distinguish these two “subspecies” are completely within the variation range of *P.sericea*. I examined a specimen from Drholec (southern Moravia Czech Republic) leg. et det. Bechyné as *P.discolorkratochvoli* Beychné, 1945, ex coll. Roubal (SNMC) which is unambiguously *P.sericea*.

Plateumarisdiscolorkratochviliformaisocoelicolor, also the forma isocupraria, forma isolacordairei, forma isopurpuricena and forma isoviolacea were described by Bechyné (1945) based on a series of specimens from Přybyslav (central Bohemia) collected by Bechyné. All these form names are infrasubspecific.

Perris (1864) described *Donacialacordairii* based on a specimen from Spain (Geiser and Geiser 2023). He allocated it to the same group (“dans la mème division”) as *Donaciasericea*. It was later regarded as an aberration of *P.discolor* (Winkler 1930; Balthasar 1934) or as a variation or subspecies *P.discolorlacordairii* (Silfverberg 2010). Askevold (1991) examined the endophallus of specimens from Spain which are assignable to *P.lacordairii*. He found it typical for *P.sericea* from other regions and therefore synonymised it with this species. I examined 31 specimens from BMNH and I agree.

*Plateumarislevigata* was described by Csiki in 1953 as an aberration of *P.sericea*.

He wrote: “*Plateumarissericea* ab[erratio]. *levigata*[sic!] nom[en]. nov[um]. pro *violacea* Gyll. (nec Pall., nec Hoppe)“. *Plateumaris* “*levigata*” is not a spelling error although “*laevigata*” is more common, but both spellings were used in classical Latin for the word “smoothed”, so “*levigata*” is correct. Anyway, this is an infrasubspecific name.

*Plateumarismicans* was described as *Donaciamicans* by Panzer in 1795 and not in 1796 according to Alonso-Zarazaga and Evenhuis (2017).

*Plateumarisnipponensis* was described by Nakane (1963) from Kamikochi, Nagano (Japan). He assigned it closely to *P.sericea* but listed several relative characters (“more shining”, “relatively shorter”) which fit easily in the variability of *P.sericea*. It is regarded as subspecies in Warchałowski (2010) and as a synonym in Hayashi (2020) to *P.sericea*.

*Plateumarisnymphaeae*: Fabricius (1792) spelled *Donacianympheae* in his original description, but this original spelling was an inadvertent error. According to ICZN 1999 (Art. 32.5.1) it has to be spelled *nymphaeae* as it was applied in Silfverberg (2010).

*Plateumarisobsoleta* Jacobson, 1894: questionable synonym, see below in *P.shirahatai*.

*Plateumarispalustris* was described as *Donaciapalustris* by Schilling in 1838 on page 99 and not in 1837 on page 104. It is a homonym because Herbst (1784) described a *Donaciapalustris* which is now synonymous with *P.bracata*.

Plateumarisdiscolorkratochviliformapseudoviolacea and forma purpuricena were described by Bechyné (1945) based on a series of specimens from Přybyslav (central Bohemia) collected by Bechyné. All these form names are infrasubspecific.

*Plateumarissibirica* (Solsky, 1871), confirmed synonym, see below.

*Plateumarissocia* was described by Chen (1941) based on three specimens from Chekiang (Zhejiang). In Silfverberg (2010) it is stated as a synonym to *P.sericeasibirica*. Askevold (1991) considered it a probable new synonym and Hayashi (2020) a synonym of *P.sericea*. The description of Chen (1941) mentions only characters which are clearly within the variation range of *P.sericea*, to which this species “is very closely allied”. It is also interesting that Gressitt and Kimoto (1961) mentioned in their key of the Chinese species not *P.sericea* (which occurs in China) but *P.socia*, separated from the other Chinese *Plateumaris* species by typical characters of *P.sericea*.

*Plateumarissericeaslovacica* Balthasar: In the coll. generalis in SNMC two specimens are stored which are labelled “Čeklís, Slovensko, *Plateumarissericeaslovacica*det. V. Balthasar, Typus n[ova]. ssp.” which I identified as *P.sericea*. I assume that Vladimir Balthasar (1897–1978), who described other species and subspecies of *Plateumaris* (Balthasar 1934) intended to describe these specimens as a new subspecies but never did. Therefore, *P.sericeaslovacica* is a nomen nudum.

Plateumarisdiscolorab.tatrica was described by Balthasar (1934) based on one or several specimens (the number is unclear) characterised by a dark purple pronotum and dark violet-blue elytra, collected by Al. Procházka, from Štrbské pleso, High Tatras, Slovakia. Anyway, this is an infrasubspecific name.

*Plateumarisviolacea* was described by Hoppe (1795) as *Donaciaviolacea*, but this is a homonym to *Plateumarisviolacea* (Pallas, 1773), originally described as *Lepturaviolacea*, which is synonym with *P.bracata*.

Csiki described 1953 “*Plateumarissericea* ab[erratio]. *viridis* nom[en]. nov[um]. pro *micans* Panz. (nec Hoppe)“, but that does not matter because this is an infrasubspecific name.

###### General remarks on the morphology and distribution of *Plateumarissericea*.

*Plateumarissericea* has the largest distribution area of all *Plateumaris* species. It is no surprise that it is also genetically very variable (Hendrich et al. 2015), which is shown also in the variability of the morphological characters. Additionally, to the colour variations mentioned above, *P.sericea* also varies in the shape and microstructure of the pronotum. While many Donaciinae species can be characterised by a typical shape of the tooth on the metafemur, *P.sericea* can show no tooth at all, or a very prominent sharp tooth and all shapes in between. Even the aedeagus varies in shape.

###### Diagnosis.

Legs and antennae usually entirely metallic, some specimens with reddish parts near the joints; pygidium of females rounded, in some specimens slightly emarginate, that of males emarginate; apex of median ejaculatory guide rounded.

###### Description.

***Size***: 6.5–10.5 mm.

***Colour***: *Plateumarissericea* shows the greatest colour variety among all Donaciinae species: The whole beetle can be bronze, green, blue, black, cupreous, purple, red, yellow, and all shades in between. Antennae and legs are mostly completely metallic, but there are some specimens with red base of the antennomeres and even with red parts of the legs, usually at the tibiae or tarsomeres.

***Head***: Same colour as pronotum, supraocular furrow indistinct; vertex with a median line, antennomeres always apically darkened, either completely dark metallic or the basal part reddish to varying degrees, A3 slightly longer than A2, A4 2× as long as A2 in most specimens. A3 ≥ A2, A4 = 2× A2.

***Pronotum***: Outline more or less quadrate, in some specimens longer than wide; anterolateral tubercles prominent but sometimes flattened, the disc varies from alutaceous and impunctate to finely or coarsely punctate with deep transverse wrinkles, the median line can be clear and deep or only a very shallow furrow.

***Elytra***: Disc rugose, rows of punctures, shape, and apex typical like in other *Plateumaris* species.

***Legs***: Entirely metallic and same colour as upper side. Rarely, some specimens show reddish parts near the joints, mostly on the tibiae or tarsomeres, metafemora of most specimens with a prominent, blade-like tooth, but some specimens with an indistinct or without any tooth.

***Pygidium***: Emarginate in males, usually rounded but sometimes shallowly emarginate in females.

***Aedeagus***: Examples of its variability are shown in Fig. 11D, E.

There are no reliable external characters to distinguish *P.sericea* from *P.shirahatai*. The only reliable feature can be found at the endophallus. The apex of the median ejaculatory guide of the endophallus is notched in *P.shirahatai* (Fig. 12B) whereas it is rounded (Fig. 12A) in *P.sericea*. The habitus of *P.sericea* also looks very similar to *P.roscida*, but the latter always has large red parts on the legs and antennae, and their aedeagi are strikingly different (Figs 9, 11D, E).

###### Biology.

*Plateumarissericea* feeds on *Carex* sp., *Juncus* sp., *Eriophorum* sp., *Scirpus* sp. and related plant species. For details and identification of the larvae see Steinhausen (1994), Narita (2003) and Bieńkowski and Orlova-Bieńkowskaja (2004). *Plateumarissericea* is the most common *Plateumaris* species and can be found in many wetland habitats throughout the Palaearctic region. It tolerates a broad range of ecological conditions if it is only wet enough.

###### Distribution.

*Plateumarissericea* has not only the largest distribution area of all *Plateumaris* species but also of all Donaciinae species. It occurs in the whole Palaearctic region. Any lack of records in some parts of its area is most probably due to a lack of collection trips there. Records exist for Europe: Austria, Belgium, Bosnia-Herzegovina [new in PalCat], Belarus, Bulgaria, Croatia [first record], Czech Republic, Denmark, Estonia, Finland, France, Germany, Great Britain, Greece [first record], Hungary, Ireland, Italy, Latvia, Liechtenstein, Lithuania, Luxembourg, Crimea [first record], Montenegro [first records], The Netherlands, North Macedonia [first record], Norway, Poland, Romania, Russia (north, central, and south parts of European territory), Serbia [new in PalCat], Slovakia, Slovenia, Spain, Sweden, Switzerland, Turkey [new in PalCat], Ukraine.

North Africa: Algeria [new in PalCat].

Asia: Armenia, Azerbaijan, China (Beijing, Hebei, Zhejiang), Georgia, Iran, Japan, Kazakhstan, Mongolia, North Korea [new in PalCat], Russia (west, east, and south Siberia [new in PalCat], Far East), South Korea [new in PalCat], Turkey [new in PalCat].

###### New country records additional to Silfverberg (2010).

Bosnia-Herzegovina: Mohr (1966b) and further new records:

Bosnia • 1 ex,; Livno [north of Buško Jezero]; collected from *Cladiummariscus*; *Donaciaimitatrix* Apfelbeck [V. Apfelbeck det.], *Plateumarissericea* E. Geiser 2019 det.; HNHM • 2 ex.; Jezero near Jaice; on *Cladiummariscus*; *Donaciaimitatrix* Apfelbeck [V. Apfelbeck det.], *Plateumarissericea* E. Geiser 2019 det.; HNHM • 1 ♀; Jezero; 1902; Apfelbeck leg.; *Plateumarisintermedia* V. Apfelbeck det., *Plateumarissericea* E. Geiser 2019 det.; NHMB [ex coll. L. Weber in coll. Frey] • 1 ex.; Alps [Dinaric Alps]; Tomov det., E. Geiser 2019 vid.; HNHM • 1 ex.; Vrelo Bosne [spring of the Bosna river, in Ilidža, west of Sarajevo]; *Plateumarisdiscolor* Apfelbeck [det.], *Plateumarissericea* I.K. Lopatin det.; HNHM • 1 ex.; Sarajevo, Igman Planina [Igman mountain west of Sarajevo]; 9 May 1930; Dr. J. Fodor leg., J. Bezděk 2017 det.; HNHM.

Croatia • 1 ex.; Pakrac [town in western Slavonia]; Plateumarisdiscolorab.lacordairii Z. Kaszab det., *Plateumarissericea* E. Geiser 2020 det., HNHM [ex coll. Apfelbeck] • 5 ex.; Plitvice [Plitvice Lakes National Park]; May 1970; [each with a different colour]; E. Geiser 2021 det.; ZFMK [coll. Prof. H. Bick].

Crimea • 1 ex.; Sebastopol; W. Pliginsky [leg.?]; Plateumarisdiscolorab.lacordairii W. Balthasar [det.?], *Plateumarissericea* E. Geiser 2020 det.; SMNC. Remark: Balthasar (1934) published a small key where he described P.discolorab.tatrica to distinguish it from P.discolorab.lacordairii. This is most likely the specimen he examined for this key because it shows exactly the same characters that he mentioned there.

Greece • 1 ex.; Thessalia, Pindos mountains, Dessi, Kalambaka, Pertouli, 1110 m; 23. May 2001; A. & F. Riedel leg.; E. Geiser 2023 det; SMNS.

Montenegro • 1 ex.; Crna Gora, Žabljak; 18. Jul 1934; Dr. J. Fodor leg.; J. Bezděk 2017 det.; HNHM • 7 ex.; Žabljak; 4 Jul. 1983; W. Grosser leg.; E. Geiser 2021 det.; BMNH [ex coll. Voříšek].

North Makedonia • 6 ex.; Delčevo; 3 Jun. 1982; I. Rozner leg.; J. Bezděk 2017 det.; HNHM.

Serbia: Gavrilovic and Curcic (2011).

Turkey: Many records from European and Asian territory in Ekiz et al. (2020).

Algeria: Goecke 1957b.

North Korea: Cho and An (2020).

South Korea: Cho and An (2020).

Russia (South Siberia): Gus’kova et al. (2018).

###### Material examined.

More than 500 specimens from different localities, labelled as various species or subspecies throughout the distribution area.

##### Plateumaris caucasica

Taxon classificationAnimaliaColeopteraChrysomelidae

Zaitzev, 1930 syn. nov.

AFDC93ED-6C5C-53FD-A8F9-0F84A0EA8B87

###### Type locality.

Russia: Stavropol and Dagestan.

###### Type material.

***Type series***: Russia • 4 ex; Ciscaucasia, Stavropol; Apr 1905; DM Maljuzhenko leg.; Russia • 5 ex; Daghestan, Chasav-jurt; E. Koenig leg.

###### Remarks.

According to Zaitzev (1930) these specimens were stored in the “Collection of the Georgian Museum”. The currently depository is unknown.

Geiser (in press) and Geiser and Bezděk (in press) treated *P.caucasica* as a synonym of *P.sericea* “based on study of comparative material, descriptions, and of biogeographical research“. Zaitzev (1930) described a new species *Plateumariscaucasica* (see Geiser and Geiser 2023) based on reddish parts of the antennomeres and legs. Additionally, he stated as different characters: “a more rugose pronotum (almost like *P.discolor*)” and “compared with *P.discolor* more slender antennae, the fourth antennomere which is twice as large as the second”. Also, he stated, it is a “intermediate species between *sericea* L. and *discolor* Panz.”

As I explained in “General remarks on synonyms of *Plateumarissericea*” above, this is a typical example of establishing a new “species” on colour characters. The other mentioned “different” characters are completely within the variation range of *P.sericea* or characteristic of this species. The morphology of the aedeagus is also completely within the variation range of *P.sericea*. In the same area also typical *P.sericea* (that is: with completely metallic antennae and legs) could be found, the colour variation form “*P.sericeacaucasica*” could not even be a subspecies.

Zaitzev assumed that *Plateumariscaucasica* is also “very close” to *P.annularis*, because both have a red base at their antennomeres and legs which are partly reddish brown. To the credit of Zaitzev it is necessary to mention that he had doubts if *P.caucasica* is really a new species or a synonym to *P.annularis*. He suggested to treat it as a new species until further knowledge is available about the East Siberian *Plateumaris* species.

*Plateumarisannularis* was synonymised by Kolossow (1930) with *P.roscida* (see there for details). Askevold (1991) suggested that “*Donaciacaucasica* (Zaitzev) (1930: 11)” [sic! it was described it as *Plateumaris* and not as *Donacia*] is a “possible new synonym” to *P.roscida*. He argued that both have the red base of the antennomeres and the description of Zaitzev agrees well with specimens of *P.roscida*, but he had doubts because *P.roscida* is known only from Asia east of lake Baikal whereas *P.caucasica* only occurs in the Caucasus region.

First, it is actually biogeographically implausible that these two species should be synonyms. Second, the pygidium is emarginate in *P.roscida* and not emarginate in *P.caucasica* in both sexes. Third, the aedeagi of *P.roscida* and *P.caucasica* are strikingly different. For the median lobe of *P.roscida* see Fig. 9. The median lobe of *P.caucasica* fits well into the variation range of *P.sericea* (Fig. 11D, E). Therefore *P.caucasica* is a synonym of *P.sericea*.

Bieńkowski (2014) stated in his identification key at *P.sericea*: The taxonomic status of the subspecies caucasica and *sibirica* needs further studies. This has been done here for *caucasica* and *sibirica* (see below).

###### Material examined.

More than 30 specimens from the Caucasus region (north and south) labelled as “*P.caucasica*”, “*P.sericeacaucasica*” or “*P.roscida*” which were all clearly *P.sericea*.

##### Plateumaris discolor

Taxon classificationAnimaliaColeopteraChrysomelidae

(Panzer, 1795)

E468C23E-4A53-58F1-9C1F-D3B2AC72E89D

###### Type location.

Germany.

###### Type material.

The holotype is unknown.

###### Remarks.

*Plateumarisdiscolor* was described by Panzer (1795) as *Donaciadiscolor* (Geiser and Geiser 2023), but the morphological variability of *P.discolor* is within the range of the variability of *P.sericea*. It was finally synonymised with *P.sericea* by Askevold (1991) by examination of the endophalli from *P.discolor* and *P.sericea* specimens which showed constant characters throughout their distribution area (Fig. 12a), but some authors continue to regard *P.discolor* as a species propria (Silfverberg 2010; Bieńkowski 2014; Rheinheimer and Hassler 2018); therefore, further arguments are discussed below.

Several characters are used to distinguish *P.discolor* from *P.sericea*. The first are the antennomeres: in *P.discolor* A3 and A4 are a little bit longer than A2, whereas in *P.sericea* A3 is 1.5× as long as A2 and A4 is twice as long as A2. In fact, the length of the antennomeres is very variable, therefore the difference between “a little bit” and “one and a half” is not clear.

The second is the pronotum disc: in *P.discolor* it is more punctured and transversely wrinkled whereas in *P.sericea* it is very finely sculptured. However, the structure of the pronotum disc varies in both “species” in its sculpture and shows an intermediary form in many cases.

The third is the median lobe of the aedeagus, which is also very variable (Fig. 11D, E). This is shown also in the drawings and pictures in identification keys. Sometimes the aedeagus picture of *P.discolor* in one key looks most similar to the picture of the aedeagus of *P.sericea* in another key. When a drawing or photograph was made from different angles of view, the same aedeagus can look different in shape and contour. There exist specimens with “*discolor*” antennomeres and “*sericea*” pronotum and vice versa. Also, each shape of the aedeagus can occur with any combination of the antennomere or pronotum characters.

Due to these variations, there are no reliable morphological characters to distinguish *P.discolor* from *P.sericea*. Other evidence suggests that they may be separate species: *P.discolor* is reported to be assigned to acid soil and peat bogs where the larvae develop on *Carex*, *Juncus* and related plants, whereas *P.sericea* prefers various wetland habitats with alkaline soil (Rheinheimer and Hassler 2018). Their larvae feeds on *Sparganium* sp. and *Irispseudacorus* (Bienkowsky, 2014). However, *P.sericea* has such a large distribution area and is very abundant even nowadays in contradiction to almost all other Donaciinae species, therefore, it is more likely that the food plant is also widespread and abundant. This is the case with *Carex* or *Juncus* but not with *Sparganium* and *Iris*. In the key to Donaciinae larvae in Japan Narita (2003) mentions *Carexdispalata* Boott. and *Scirpusfluviatilis* (Torr.) A. Gray as food plants for the larvae of *P.sericea*. This is definitely not a confusion with *P.discolor* because the latter does not occur in Japan. Therefore, the assignment to the food plants in Bienkowsky (2014) contradicts the study of Narita (2003) and is in general not a suitable argument that *P.discolor* is a separate species.

Molecular studies by Hendrich et al. (2015) and J. Bergsten (pers. comm. NHRS, 23 Jan 2023) indicate that *P.sericea* is genetically very variable. In molecular phylogenetic trees, specimens identified as *P.discolor* are resolved in between *P.sericea* specimens, sometimes in groups and separated from *P.sericea* groups, sometimes not. It is likely that some of these specimens identified as *P.sericea* are “some kind of” *P.discolor* and vice versa, because morphological characters are not reliable to distinguish them. There is another problem: it is possible that *P.sericea* consists of several cryptic species but *P.discolor* may not be one of them.

###### Material examined.

More than 100 specimens labelled “*P.discolor*” from different localities in Europe.

##### Plateumaris sibirica

Taxon classificationAnimaliaColeopteraChrysomelidae

(Solsky, 1871)

7B16BDCB-185C-5540-A387-613A6C792101

###### Type locality.

Russia, Irkutsk.

###### Type material.

Solsky (1871) did not indicate the depository. It is unknown if the holotype still exists.

###### Remarks.

*Plateumarissibirica* was described as *Donaciasibirica* by Solsky (1871) as a new species from Irkutsk which resembles *P.sericea* (Geiser and Geiser 2023). It was not described as a “variation” as is sometimes cited in the literature. Jacoby (1885: 193) doubted it: “Donaciasericea var. sibirica? Solsky: The dozen specimens obtained at Nikko show scarcely any difference from our European form … Structural differences I can see none.”

Eventually, the original description only mentions characters which are typical for *P.sericea*. It has been regarded as a synonym to *P.sericea* by Goecke (1960) and Hayashi (2020), but it is treated as a subspecies in Silfverberg (2010), in Warchałowski (2010), and in Bieńkowski (2014). The latter mentioned that “the taxonomic status of the subspecies *P.sericeasibirica* needs further studies”.

I examined more than 60 specimens identified as *P.sericeasibirica*, mainly from the BMNH, NHMB, NMPC and SDEI, and I agree with Jacoby, Goecke and Hayashi that all characters are clearly within the variation range of *P.sericea*. I could not find any differences compared with European or other Siberian specimens. Therefore, I confirm the decision of Goecke (1960) and Hayashi (2020) that *P.sibirica* is neither a valid species nor a subspecies, but synonym of *P.sericea*.

The original description was mostly cited as Solsky (1872). It was described in “Horae Societatis Entomologicae Rossiae” volume 8 comprising the years 1871 and 1872. There it was described in the part of 1871 according to Standfuss and Kerzhner (2004).

##### Plateumaris shirahatai

Taxon classificationAnimaliaColeopteraChrysomelidae

Kimoto, 1971

239F4F88-114B-5939-BA4F-42ED8639E875

Fig. 13

Plateumaris shirahatai Kimoto, 1971: 1.
Plateumaris macropenis Nakane, 1999: 45.
? Plateumarisobsoleta Jacobson, 1894.

###### Type localities.

*Plateumarisshirahatai*: Japan, Honshu, Yamagata Prefecture, Shizu, Gassan; *Plateumarismacropenis*: Japan, Honshu, Oze.

###### Type material.

***Holotype*** of *P.shirahatai*: Japan • 1♂, Yamagata Prefecture, Shizu, Gassan; 17 Jun 1960; K. Shirahata leg.; Entomological Laboratory, Faculty of Agriculture, Kyushu University, Fukuoka. The holotype was not examined.

***Paratype*.** Japan • 3 ♀; same data as for the holotype; Japanese Insect Collection No. 21963, OMNH.

***Holotype*** of *P.macropenis*. Japan • 1 ♀; Honshū, Oze; 15 Jul. 1950; H. Hasegawa leg.; *Plateumarismacropenis* T. Nakane det.; Laboratory of Systematic Entomology, Faculty of Agriculture, Hokkaido University, Sapporo, Japan.

###### Taxonomic history and synonymies.

*Plateumarismacropenis* Nakane, 1999 was synonymized by Hayashi and Shiyake (2004) on page 117. The holotype of *P.macropenis* is a female specimen of *P.shirahatai*.

? *Plateumarisobsoleta* Jacobson, 1894: see below.

###### Diagnosis.

Pronotal disc rugose, antennae, and legs entirely metallic, although in some specimens the basis of the antennomeres is reddish, A3 = 1.5–2× A2, tooth on metafemur sharp blade-like or obtuse, pygidium of females rounded, in some specimens slightly emarginate, pygidium of males emarginate or truncate, median process of endophallus notched.

**Figure 13.**
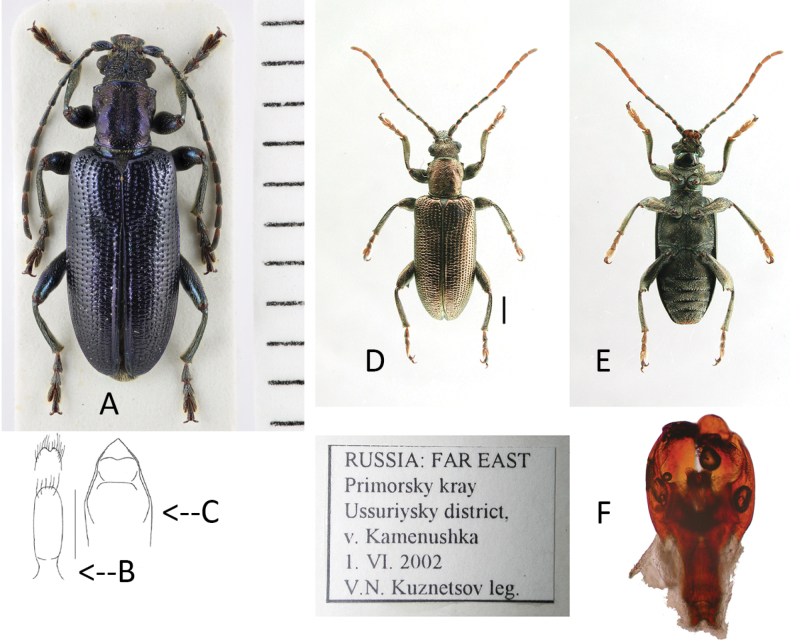
*Plateumarisshirahatai***A** habitus (photograph by K. Matsumoto) **B** cap of tegmen, shape slightly variable **C** median lobe (**B, C** from Hayashi 2020) **D, E** habitus from dorsal and ventral **F** endophallus (**D–F** photographs by M Hayashi). Scale bars: one unit – 1 mm (**A**); 0.5 mm (**B–E**).

###### Description.

***Size***: Males 6.5–7.3 mm, females 7.8–8.2 mm.

***Colour***: Upper side colour very variable: blackish, blue, green, bronze, cupreous, purple, same colours as *P.sericea*. Antennae and legs same colour as upper side, in some specimens with reddish parts near the joints.

***Head***: Rugulosely punctate and pubescent, frontal calli convex, interocular area with a longitudinal median furrow.

***Antennae***: Entirely metallic with same colour as dorsum, antennomeres in some specimens basically reddish, filiform, A1 robust, club-shaped, A1 = 2× A2, A3 ≅ 2× A2, A3 < A4 ≤ A5.

***Pronotum***: Slightly longer than broad, gradually narrowed posteriorly, dorsal surface with a pair of distinctly raised antero-lateral tubercles, and with a triangular depression medio-basally, disc punctate with transverse rugae, median line indistinct, shallowly furrowed.

***Elytra***: Interstices of the rows of punctures with close oblique or transverse corrugations and showing a rugged appearance.

***Legs***: Entirely metallic, same colour as dorsum, in some specimens small reddish parts at the base of the joints, tooth of metafemur prominent and blade-like but also in some specimens obtuse.

***Pygidium***: Apex pubescent, apical shape in females rounded, in some specimens slightly emarginate, in males emarginate or truncate.

***Male genitalia***: Median lobe of aedeagus (Fig. 13C) very similar to *P.sericea* (Fig. 11D). Cap of tegmen rounded or slightly notched (Fig. 13B). Apex of median ejaculatory guide of the endophallus notched (Figs 12B, 13F).

###### Remarks.

The only reliable feature to distinguish *P.shirahatai* from *P.sericea* is the notched apex at the median ejaculatory guide of the endophallus. The habitus of *P.shirahatai* looks also very similar to *P.roscida*, but the latter always has large red parts on the legs and antennae, and their aedeagi are remarkably different (Figs 9, 13B, C).

###### Biology.

The larvae feed on *Carex* sp. (Narita, 2003). Adults were collected on the florescence of *Carex* sp. (Hayashi and Tominaga 2005).

###### Distribution.

East Palaearctic species. The distribution area of *P.shirahatai* is situated completely within the eastern area of *P.sericea*. Unfortunately, it is almost impossible to distinguish *P.shirahatai* from *P.sericea* without male genitalia. Both species share the same colour spectrum and same variation of the other external characters.

Records exist for Asia: China (Jilin) [new in PalCat], Japan (Hokkaido and Honshu), Mongolia [new in PalCat], Russia (Far East: Primorsky Krai, Sakhalin), South Korea, South Kuril (Etorofu).

###### New country records additional to Silfverberg (2010).

China (Jilin): Hayashi (2020); Jilin Province, det. M. Hayashi (Zoological Institute, Chinese Academy of Science, Beijing).

Mongolia: • 2 ♂♂, 1 ♀; central Mongolia, Terelj; 47°59'24"N, 107°27'E; 28 Jun 2004; M. Hayashi leg.; M. Hayashi det.; Hoshizaki Institute for Wildlife Protection, Izumo, Japan. Remark: Some parts of this data are published in Hayashi (2020). The details were obtained from M. Hayashi (pers. comm. 27 May 2020).

This recent record from central Mongolia shows clearly that the distribution area is not known until now. As *P.shirahatai* was described only in 1971 it is likely that some specimens from the East Palaearctic stored in collections may be identified as *P.sericea*.

###### Material examined.

20 specimens from Hokkaido and Honshu.

##### ? Plateumaris obsoleta

Taxon classificationAnimaliaColeopteraChrysomelidae

Jacobson, 1894

A8AB1035-D216-5ECA-B50A-283437912949

Figs 14 , 15 , 16

###### Type locality.

Russia, Far East, Primorsky Krai, Bay of Posyet.

###### Type material.

**Figure 14.**
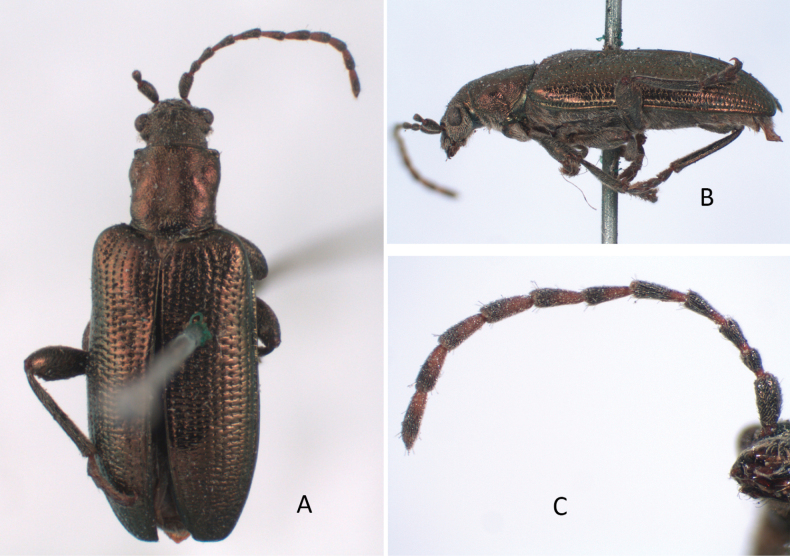
*Plateumarisobsoleta* holotype **A** habitus **B** lateral view **C** right antennae with red base of the antennomeres (photographs by A. Moseyko).

**Figure 15.**
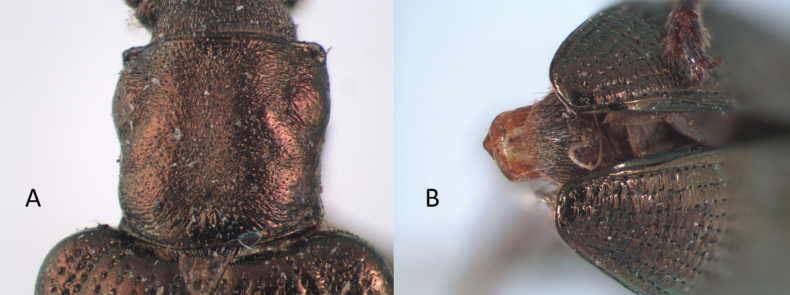
*Plateumarisobsoleta* holotype **A** pronotum **B** ovipositor protruding from abdomen (photographs by A. Moseyko).

***Holotype***: Russia • 1 ♀; Far East; Primorsky Krai; Bay of Posyet; ZIN. Only the holotype exists. It was examined from photographs only (Figs 14, 15).

###### Remarks.

At first, I intended to synonymise *P.obsoleta* with *P.sericea* based on studies of the type material and description, but doubts remained that it is more likely that *P.obsoleta* is a synonym with *P.shirahatai*. I am sure that *P.obsoleta*, described based on one female specimen and never recorded again in more than 100 years, is a synonym. However, I cannot prove if it belongs to *P.sericea* or to *P.shirahatai* because it is impossible to distinguish these two species by external morphological characters. These two species differ only by subtle morphological differences in the apical part of the endophallus (Fig. 12).

**Figure 16.**
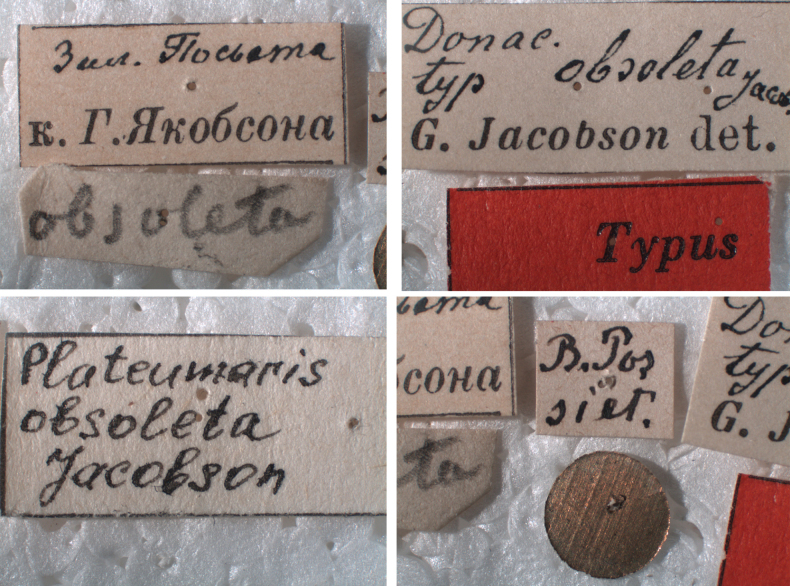
*Plateumarisobsoleta*: All labels tagged to the holotype (photographs by A. Moseyko).

*Plateumarisobsoleta* was described by Jacobson (1894) (see Geiser and Geiser 2023) based on a single specimen collected in Russia, Far East: Posyet in Primorsky Krai. No other specimen of *P.obsoleta* has been recorded in the last 130 years; it only appears regularly in identification keys. Jacobson found it most similar to *P.discolor* and *P.sericea*. All characters he described are also typical characters of *P.sericea*. Whereas many specimens of *P.sericea* have a sharp and prominent tooth at the metafemur, in some specimens this tooth can be blunt or is lacking completely. According to Jacobson (1894) this holotype is a male specimen. However, Bieńkowski (2014) wrote in his key: only one single female specimen is known. He also published four drawings of some details of this specimen. In fact, the holotype is stored in ZIN, from which I obtained some detailed photographs (Figs 14, 15, 16).

The controversy about the sex of this specimen can now be solved: the apical part of the ovipositor protrudes, which Jacobson misinterpreted as a part of the aedeagus. Although Askevold (1991) had not seen the holotype, but he suspected that the specimen described by Jacobson was female. According to the original description, “Pygidium apice rotundatum” is a description of a female specimen because no known males of species of *Plateumaris* have a rounded pygidium. All the characters described by Jacobson and the characters which could be examined on the photographs of the holotype fit easily within the variation range of *P.sericea*. However, *P.shirahatai* also occurs in southern Primorsky Kraj (Hayashi and Tominaga 2005). Photographs (Figs 14C, 15A) show that many features of *P.obsoleta* are consistent with those of *Plateumarisshirahatai* identified in Primorsky (Fig. 13D, E) including metallic legs and an indistinct median line on the pronotum. In addition, the antennae of *P.shirahatai* are variable in colouration, with some individuals having the same colouration as the type of *P.obsoleta*. This strongly supports the possibility that *P.shirahatai* is a synonym of *P.obsoleta*. On the other hand, *P.obsoleta* has a small metafemoral tooth, but it is suspected that the shape of this tooth may be malformed. This is a recurrent problem with species described on single specimen (pers. comm. M. Hayashi, 04 Apr 2023). Therefore, it seems more likely that *P.obsoleta* is synonym with *P.shirahatai* than with *P.sericea*, that was also suspected by Askevold (1991), Hayashi and Tominaga (2005), and Warchałowski (2010). The pronotum of the type specimen of *P.obsoleta* (Fig. 15A) looks similar to the pronotum of *P.shirahatai* (Fig. 13A, D). All in all, the decision of the synonymisation cannot be made now.

Perhaps it will be possible in the near future to solve this problem without destroying this single specimen with more elaborate methods than historical DNA analysis. The solutions used to extract the DNA may be destroy the connecting membranes between the chitinous parts. Nowadays, nobody can guarantee that this specimen would NOT be damaged! Additionally, it is very questionable whether the results will be clear enough. Usually, the DNA in old, stored insects is fragmented and cannot be sufficiently reconstructed to make the decision to which species the specimens belong. *Plateumarissericea* and *P.shirahatai* are closely related, which was proofed by DNA analysis (Hayashi and Sota 2014). There are only few sections of the DNA where the differences are shown. It is unlikely that exact these few sections could be tracked down by the current methods. Therefore, according to the current state of knowledge, I cite it as a “probable new synonymy”. If it once can be prooved that *P.shirahatai* and *P.obsoleta* are synonyms, the name *P.obsoleta* has priority because it was described in 1894 and *P.shirahatai* in 1971.

##### Plateumaris weisei

Taxon classificationAnimaliaColeopteraChrysomelidae

(Duvivier, 1885)

A995AB7F-7228-5275-867D-15B3E7D2E71D

Fig. 17

Donacia weisei Duvivier, 1885: cxvi.
Donacia borealis Mannerheim [nomen nudum].
Plateumaris hirashimai Kimoto, 1963: 13.
Donacia (Plateumaris) mongolica Semenov, 1895: 267.
Plateumaris morimotoi Kimoto, 1963: 13.
Plateumaris consimilis orientalis Shavrov, 1948: 49.
Plateumaris sachalinensis L. N. Medvedev, 1973: 876.

###### Type localities.

*Plateumarisweisei*: Siberia. Original label text: “Sibérie coll. Duvivier“; *Plateumarisconsimilisorientalis*: Far East, Vladivostok, Sedanka, Russia; *Plateumarishirashimai*: Hokkaido, Ashoro in Tokachi, Japan; *Plateumarismongolica*: North Mongolia, Borcha-Urga, Mongolia; *Plateumarismorimotoi*: Hokkaido, Tenninkyo Mt. Daisetsu, Japan; *Plateumarissachalinensis*: Far East, Sakhalin, Yuzhno-Sakhalinsk, Russia.

###### Type material.

Type of *Plateumarisweisei*: 1 syntype, Siberie coll. Duvivier; Museum Paris coll. H. Clavareau 1932, vid. I.S. Askevold 1984 (MNHN-EC-EC2129). Image of type specimen: https://science.mnhn.fr/institution/mnhn/collection/ec/item/ec2129?listIndex=2&listCount=6.

Type series of *P.consimilisorientalis*: Russia • 3 ♀; Far East, Vladivostok, Sedanka; 19 Jun. 1937 [present depository unknown].

Type of *P.hirashimai*: Japan • 1 ♀; Hokkaido, Ashoronuma in Tokachi; 28 Jul. 1949; R. Matsuda leg.; collection Entomological Laboratory, Faculty of Agriculture, Kyushu University, Fukuoka, Japan.

Type of *P.mongolica*: Mongolia • 1 ♂; valley of the river Borcha, from Urga to the East; 6 Jul. 1894, B. Kaschkarow leg.; collection Semenov [present depository unknown].

Type of *P.morimotoi*: Japan • 1 ♂; Hokkaido, Tenninkyo Mt. Daisetsu; 27 Jul. 1955; K. Morimoto leg.; collection Entomological Laboratory, Faculty of Agriculture, Kyushu University, Fukuoka, Japan.

Type of *P.sachalinensis*: Russia • 1 ♂; Far East, Sakhalin, Yuzhno-Sakhalinsk; 12 Jul. 1955; collection of N.N. Filippov [present depository unknown]. Paratype: Russia • 1 ♂; Far East, Sakhalin, Yuzhno-Sakhalinsk; 10 Jul. 1955; [red label:] Paratype *Plateumarissachalinensis* Medvedev, *Plateumarisweisei* Duv. E. Geiser 2021 det.; NMEG.

The photographs of the syntype of *P.weisei* and the paratype of *P.sachalinensis* were examined.

**Figure 17.**
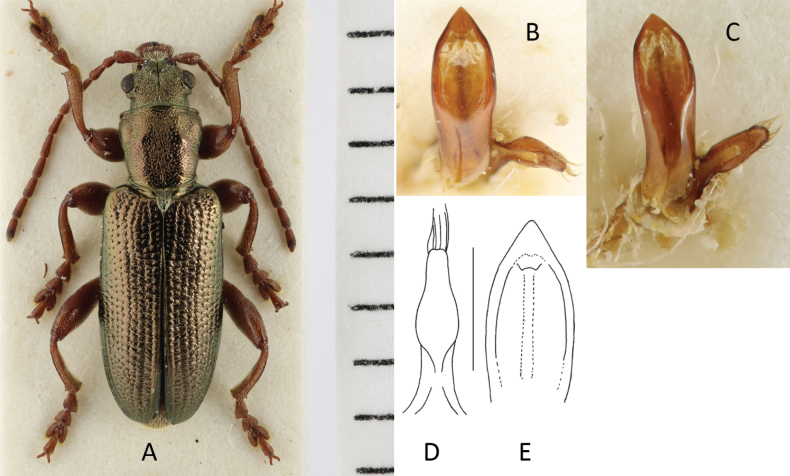
*Plateumarisweisei***A** habitus **B, C** aedeagus: Median lobe and cap of tegmen (photographs by K. Matsumoto) **D** cap of tegmen **E** median lobe (from Hayashi 2020). Scale bars: one unit – 1 mm (**A**); 0.5 mm (**D, E**).

###### Taxonomic history and synonymies.

This list of synonyms and their shifting positions (see below) indicate the main systematic problems with *P.weisei*. First, it is difficult to distinguish it from other *Plateumaris* species. The variety of conspicuous morphological characters (colour, relative length of antennomeres, shape and structure of pronotum, shape of metafemoral tooth, etc.) overlap with other species. Second, the locality name in the first description “Siberie” is anything but precise. Third, *P.weisei* has a particularly wide distribution range, from northern Fennoscandia through European Russia and Siberia to Far East, Mongolia, Northern China, the Korean peninsula, and Japan. Altogether this resulted in the new descriptions of *Plateumaris* species when a specimen was found outside Siberia with slightly different characters.

*Donaciaborealis* (Mannerheim), nomen nudum: the first who recognised that a specimen of still undescribed *Plateumarisweisei* belonged to a new species was Carl Gustav Mannerheim, a Finnish entomologist (1797–1854). He labelled a specimen (possibly more than one specimen, but I only found this one) from “Lapponica” with “*Donaciaborealis*”, which clearly is *P.weisei* (vid. E. Geiser 20 Jul. 2022). This specimen is stored in the coll. Mannerheim (LUOMUS). Mannerheim had the intention to describe it, but he died before he could publish a description.

*Plateumarishirashimai* was first described by Kimoto (1963) from Japan, Hokkaido. Askevold (1991: 58) synonymised it with the statement “The endophallus of specimens of *P.hirashimai* is indistinguishable from that of specimens of *P.weisei* from Finland in any significant way nor do they differ significantly in external structure“.

*Donaciamongolica* was described by Semenov (1895) based on a single male specimen from Mongolia, east of Ulaanbaatar. He also regarded *Plateumaris* as a subgenus to *Donacia* where this new species should be allocated. The description is very detailed (Geiser and Geiser 2023). Additionally, Semenov listed many characters to distinguish the new species from *P.consimilis*, *P.rustica* and *P.weisei*. Nevertheless, he suspected that this new species could be an aberration of *P.weisei*, which he had never seen then. Askevold (1991: 58) checked the description and suggested that *P.mongolica* is probably a synonym of *P.weisei*. It is regarded as a genuine synonym by Hayashi (2001, 2020), Warchałowski (2010) and Silfverberg (2010). I examined 9 specimens from northern and central Mongolia identified as *P.mongolica* (stored in coll. Frey in NHMB, in NMEG, and in ZFMK). They show completely yellow legs and almost completely yellow antennomere (only some distal antennomeres are darkened at the apex). Their metafemoral tooth is clearly visible but well within the variation of *P.weisei* and not so prominent as in *P.amurensis*. They all are typical *P.weisei* and *P.mongolica* is a synonym of *P.weisei*.

*Plateumarismorimotoi* was first described by Kimoto (1963) from a single male specimen from Japan Hokkaido. After studying additional material Kimoto (1981: 25) concluded that *P.morimotoi* is only an infraspecific variation of *P.hirashimai* and therefore synonymised it with the latter. Then Askevold (1991: 58) synonymised *P.hirashimai* with *P.weisei* (see above). Subsequently, *P.morimotoi* became a synonym to *P.weisei*, too.

*Plateumarisconsimilisorientalis* was described by Shavrov (1948) as a new subspecies of *P.consimilis* from Vladivostok, based on three female specimens. His detailed description (Geiser and Geiser 2023) fits to *P.consimilis* as well as *P.weisei*. He also discussed the controverse opinion of Kolossow (1930) that *P.consimilis* is distributed only in the western Palaearctic, whereas Reitter indicated “Europa, Sibirien, Japan”. For Shavrov this new subspecies was a proof or a very strong likelihood that *P.consimilis* occurred in the whole Palaearctic region. He also recognised that some features are different from the European specimens, but he deduced that such differences are due to the huge distance. Therefore, separate subspecies of European species are common in beetles of the Far East.

Askevold (1991: 58) assessed *P.consimilisorientalis* as a “probable new synonymy” by studying the original description. However, he also considered *P.amurensis* as synonym to *P.weisei*. Therefore, it is not clear, to which of these two species it is synonym because *P.amurensis* also occurs in the same area. Hayashi (2001) studied *P.weisei* and *P.amurensis* thoroughly and worked out that these are unambiguously two different species. *Plateumarisamurensis* has (mostly!) a prominent, blade like metafemoral tooth whereas *P.consimilisorientalis* has no metafemoral tooth or only a slight protrusion. He also listed *P.consimilisorientalis* as synonym with *P.weisei*.

In the coll. Frey (NHMB) I found two specimens from Japan, Honshu, Fukushima, labelled “*Plateumarisconsimilis* Schrank det. M. Chȗjȏ”, both collected in 1948. These two specimens refer not to *P.consimilisorientalis* Shavrov but were only misidentifications of *P.constricticollis*. At this time, the distribution area of *P.consimilis* was regarded to reach as far as Japan. I never saw a specimen from East of Ural which had some similarity with *P.consimilis*.

*Plateumarissachalinensis* was described by Medvedev (1973) as a new *Plateumaris* species from the Sakhalin Island (Geiser and Geiser 2023). He compared it with *P.weisei* in some characters (long antennae, metaformal tooth very weak) but put it close to *P.obsoleta* (which is synonymous with *P.sericea* or *P.shirahatai*). He regarded it as an intermediate form between the *P.weisei* and *P.amurensis* group and the *P.sericea* group. Later, Medvedev (1978) even regarded *P.sachalinensis* as a synonym of *P.obsoleta*. I studied the paratype specimen from NMEG: In contrast to many *P.weisei* specimens which have reddish antennae and legs, in this specimen large parts of the legs are metallic darkened. Probably, this colouration of the legs prompted Medvedev (1973) to place this species close to *P.obsoleta*. Also, the apical part of each antennomere is darkened. This and the other characters fit easily into the range of variability shown by *P.weisei* (for more morphological details see Hayashi 2001).

After the study of the original description Askevold (1991: 58) suggested that *P.sachalinensis* should be regarded as a “probable new synonymy” to *P.weisei*. Hayashi (2001, 2020) and Silfverberg (2010) regarded it as a synonym, Bieńkowski (2014) considered it as a valid species. Warchałowski (2010) separated it in his key from other *Plateumaris* species because of the dark metallic legs but also mentioned that it is regarded as synonym to *P.weisei* by some authors. Although Askevold (1991) regarded *P.amurensis* as synonym to *P.weisei*, the weak metafemoral tooth of *P.sachalinensis* is a typical character of *P.weisei* and excludes *P.amurensis* here. Hayashi (2001), who finally separated *P.weisei* and *P.amurensis*, confirmed the synonymy of *P.sachalinensis* with *P.weisei*.

###### Diagnosis.

Pronotal disc finely rugose and punctured, sometimes with microsculpture, median line obsolete, sometimes shallowly furrowed (similar to *P.shirahatai*), metafemur with a small, not blade-like tooth, usually rufous at the base; aedeagus with apex of median lobe arced on both sides, gradually narrowed apically, without a median lip.

###### Description.

***Size***: Males 6.2–7.0 mm, females 6.8–8.0 mm.

***Colour***: Most specimens dorsally cupreous or bronze, sometimes metallic green, blue, purple, or non-metallic brown.

***Head***: Eyes small, supraocular furrow indistinct, vertex pubescent with deep median line, antenna entirely rufous in most specimens but in some specimens darkly rufous or apically metallic, antennomeres: A5 longest in second to A6 and ca 3.5× as long as wide, A4 ca 2.2× as long as A2.

***Pronotum***: Outline subquadrate, slightly longer than wide, basal part narrowed, slightly cone-shaped, anterior tubercles distinctly visible or almost entirely smooth, disc more or less punctate, rugulose, median groove indistinct or shallowly furrowed.

***Elytra***: Transverse rugae between the rows of punctures, especially on interstices 1–4.

***Legs***: Yellow-reddish, in some specimens more or less darkened, femur, tibia, and tarsomere pubescent, outer apical angles of pro- and mesotibiae with a spine, outer apical angles of metatibiae with a small spine, metafemoral tooth mostly blunt or moderate.

***Aedeagus***: Apex of median lobe arced on both sides, gradually narrowed apically, without a median lip, cap of tegmen rounded at apex (Fig. 17).

The main different features between *P.amurensis* and *P.weisei* are shown in Table 3. The east Palaearctic *Plateumaris* species are not easy to distinguish which was also explained in the comments to the synonyms. Misidentifications are common. Oddly enough, in several collections I found the label “*Plateumaris* [or *Donacia*] *weisei* Duvivier” attached to blue specimens from central Asia, collected circa 1900, which in fact were *Donaciabactriana* Weise, 1887. Somehow the author’s name has been shifted and was then regarded as the species name.

###### Biology.

Larvae were found at the roots on *Carex* sp. (Bieńkowski and Orlova-Bieńkowskaja 2004; An 2019). Narita (2003) described the last instar of the larvae which he gained from the host plant *Carexmiddendorfii*.

###### Distribution.

*Plateumarisweisei* is a Trans Palaearctic species, it occurs from northern Fennoscandia through Siberia to the Far East, northern China, the Korean peninsula, and Japan. Also, it occurs in a broad span of latitudes, from the arctic polar circle (67°N) to 35°N in Korea. Records exist for Europe: Sweden, Finland, Russia (north and central part of European Russia).

Asia: China (Heilongjiang [new in PalCat], Inner Mongolia), Japan (Hokkaido), Mongolia, Russia (west, east, and south Siberia [new in PalCat], Far East), South Korea [new in PalCat].

In Japan records exist only from Hokkaido so far (Hayashi 2020), whereas fossil and subfossil records are known from Honshu and Kyushu, too (Hayashi and Shiyake 2011).

###### New country records additional to Silfverberg (2010).

China • 1 ex.; Heilongjiang, “Manchuria“ Harbin; *Plateumarisweisei* E. Geiser 2020 det.; SDEI [coll. K.-H. Mohr]. Remarks: Silfverberg (2010) recorded *P.weisei* for China with “NE” because the specimens are labelled only with the locality “Manchuria”, a historical region in northeast China. NE China today comprises the provinces of Heilongjiang and Jilin, and Harbin belongs to Heilongjiang. I examined 8 of these specimens stored in BMNH, in coll. Frey in NHMB, and in SDEI.

Russia • 5 ex.; South Siberia, Angara (near Baikal); I. Askevold 1985 det., E. Geiser 2019 vid.; Sharp-coll. 1905 – 313, BMNH; Bieńkowski (2014).

South Korea: Hayashi and Cho (2017); An (2019).

###### Material examined.

More than 80 specimens from Europe and Asia.

## Discussion

The changes in Geiser (in press) compared with the statuses in Silfverberg (2010) concerned mostly synonymies and country records for Palaearctic *Plateumaris* species. Forty-one countries or parts of countries could be added to the lists. These records are due mostly to faunistic publications since 2010. Many faunistic studies of small areas have been published in respective local languages and can be easily overlooked. Thanks to colleagues who provided me with such papers, many of these records could now be evaluated. Museum studies and personal communications from colleagues also provided many unpublished records. Thirteen of them were first country records and many others were confirmations of the occurrence of a specific species in these countries.

A primary objective was to declutter doubtful species and ambiguous synonymisations to answer these questions: how many species of *Plateumaris* exist in the Palaearctic region, what are their names, and how should the remaining 70 names used in a description for a *Plateumaris* taxon be allocated to the valid species? This was successful in most cases. Additionally, some holotypes could be tracked down, e.g., of *P.tenuicornis*, which could be identified unambiguously as synonymous with *P.consimilis*. This also confirmed Bechyné’s (1942) opinion that had been ignored. *Plateumarissulcifrons*, often allocated to the wrong country, could now be identified as *P.rustica* by the detailed and clear first description.

In general, this study largely confirmed the systematic status published by Askevold (1991). He stated nine valid species of Palaearctic *Plateumaris* and I agree with eight of them: his ninth species is *P.weisei*, which he thought to be synonymous with *P.amurensis*. Later Hayashi (2001) concluded that *P.amurensis* is in fact a valid species, so there are ten species in the genus. I also agree with the opinions of Hayashi and Sota (2014) and Hayashi (2020) about the valid species and their synonyms for *Plateumaris*.

Many problems could be solved unambiguously, but not all *P.obsoleta* may be a synonym of *P.shirahatai*: it could in fact be a synonym of *P.sericea*, which also occurs at the same locality and cannot be distinguished from *P.shirahatai* by external characters alone.

A problem does remain concerning the synonymisation of *P.discolor* with *P.sericea*. They are not distinguishable morphologically, even if some identification keys suggest that they are. The allocations to their ecological requirements and food plants are contradictory: because *P.sericea* shows very high genetic variability and has a very large distribution area, it may consist of cryptic species, possibly indicating evolution in progress.

I am reluctant to designate neotypes at this point. Before choosing specimens as neotypes one should be certain that the holotype or type series do not exist. Some museum collections, where I expect to find a missing holotype, could not be visited in the last years due to Covid 19, but this should be rectified in the near future. Additionally, the specimens for neotypes should be from the same location or as near as possible to where the original type or type series were collected, and this require visits to museums in which such specimens are stored.

Finally, I do not want to sweep the most severe problem in researching Palaearctic *Plateumaris* species under the carpet: the majority of specimens for systematic studies is stored in museums. It is not very hopeful to try to catch specimens in the field. Most museum specimens are by-catches acquired by luck: there exists specimens from expeditions which took place 100 and more years ago from large areas of Asia. From areas like Syria, Afghanistan, and large parts of China such Xinjiang Province, the only information we can access now is from museum specimens. The application of historical DNA methods seems to be helpful but is problematic: its success is questionable, mostly due to the severe fragmentation of the DNA in old specimens. Also, damaging these few very precious specimens by the extraction solution is likely. Perhaps, in the future, there will be gentler methods developed for such studies.

Even without restrictions for field studies caused by politics, in Europe, too, it is difficult to obtain new samples. Many colleagues who know that I am working on Donaciinae have tried to catch specimens during their own field trips in the last years. Besides *P.sericea*, all other species are rare because of many changes of the limnic environment during the last 100 years. Water pollution is not such an issue as it was 40 years ago, but man-made changes to diverse limnic habitats, especially bank straightening and drainage, has probably led to the extinction of *Plateumaris* populations that need specific ecological conditions to survive and thrive.

## Supplementary Material

XML Treatment for Plateumaris

XML Treatment for Plateumaris akiensis

XML Treatment for Plateumaris amurensis

XML Treatment for Plateumaris bracata

XML Treatment for Plateumaris consimilis

XML Treatment for Plateumaris tenuicornis

XML Treatment for Plateumaris constricticollis

XML Treatment for Plateumaris roscida

XML Treatment for Plateumaris rustica

XML Treatment for Plateumaris sulcifrons

XML Treatment for Plateumaris sericea

XML Treatment for Plateumaris caucasica

XML Treatment for Plateumaris discolor

XML Treatment for Plateumaris sibirica

XML Treatment for Plateumaris shirahatai

XML Treatment for Plateumaris obsoleta

XML Treatment for Plateumaris weisei

## Additional information

### Author ORCIDs

Elisabeth Geiser https://orcid.org/0009-0005-4536-8938

### Data availability

All of the data that support the findings of this study are available in the main text.
